# New Derivatives of *N-*Hydroxybutanamide: Preparation, MMP Inhibition, Cytotoxicity, and Antitumor Activity

**DOI:** 10.3390/ijms242216360

**Published:** 2023-11-15

**Authors:** Anastasia Balakina, Svyatoslav Gadomsky, Tatyana Kokovina, Tatyana Sashenkova, Denis Mishchenko, Alexei Terentiev

**Affiliations:** 1 Federal Research Center of Problems of Chemical Physics and Medicinal Chemistry RAS, 142432 Chernogolovka, Russia; balakina@icp.ac.ru (A.B.); gadomsky@icp.ac.ru (S.G.); kota6711@yandex.ru (T.K.); tsashen52@mail.ru (T.S.); mdv@icp.ac.ru (D.M.); 2 Faculty of Fundamental Physical-Chemical Engineering of M.V. Lomonosov MSU, Leninskie Gory, 119991 Moscow, Russia; 3 Scientific and Educational Center in Chernogolovka, State University of Education, 141014 Mytishchi, Russia

**Keywords:** *N*-hydroxybutanamides, hydroxamic acids, *N*-substituted succinimide ring opening method, matrix metalloproteinases, cytotoxicity, acute toxicity, antitumor activity, antimetastatic activity

## Abstract

Using a novel method of *N*-substituted succinimide ring opening, new *N*-hydroxybutanamide derivatives were synthesized. These compounds were evaluated for their ability to inhibit matrix metalloproteinases (MMPs) and their cytotoxicity. The iodoaniline derivative of *N*^1^-hydroxy-*N*^4^-phenylbutanediamide showed the inhibition of MMP-2, MMP-9, and MMP-14 with an IC_50_ of 1–1.5 μM. All the compounds exhibited low toxicity towards carcinoma cell lines HeLa and HepG2. The iodoaniline derivative was also slightly toxic to glioma cell lines A-172 and U-251 MG. Non-cancerous FetMSC and Vero cells were found to be the least sensitive to all the compounds. In vivo studies demonstrated that the iodoaniline derivative of *N*^1^-hydroxy-*N*^4^-phenylbutanediamide had low acute toxicity. In a mouse model of B16 melanoma, this compound showed both antitumor and antimetastatic effects, with a 61.5% inhibition of tumor growth and an 88.6% inhibition of metastasis. Our findings suggest that the iodoaniline derivative of *N*^1^-hydroxy-*N*^4^-phenylbutanediamide has potential as a lead structure for the development of new MMP inhibitors. Our new synthetic approach can be a cost-effective method for the synthesis of inhibitors of metalloenzymes with promising antitumor potential.

## 1. Introduction

Hydroxamic acid (HA) derivatives are well-studied compounds with significant biological activity. Over the past century, since the discovery of HAs, extensive research has been conducted on their synthesis and biological activity. HAs have been shown to inhibit a variety of enzymes, including ureases, peroxidases, histone deacetylases, and matrix metalloproteinases. HAs have demonstrated a broad spectrum of biological activities, such as antitumor, insecticidal, and antimicrobial properties. Several hydroxamic acid derivatives have been approved for clinical use [1,2,3,4,5].

**Figure 1:**
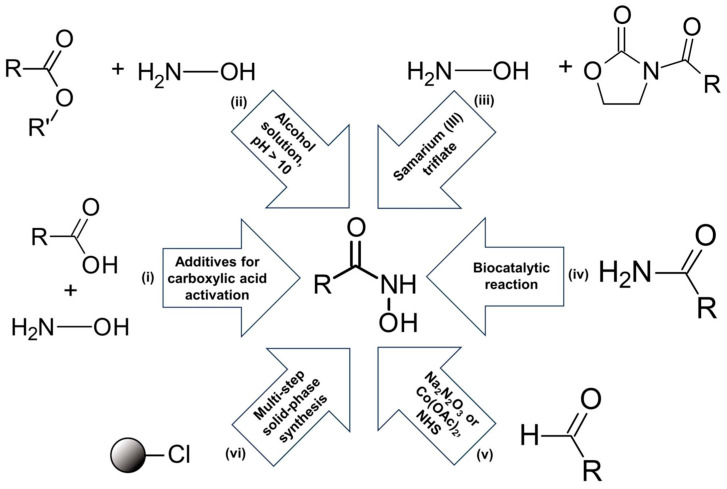
Basic approaches to the synthesis of hydroxamic acids. (i) Synthesis of HAs from carboxylic acids using their activated forms. (ii) Synthesis of HAs from esters. (iii) Synthesis of HAs from *N*-acyloxazolidinones in the presence of samarium triflate. (iv) Synthesis of HAs from amides in the presence of *Pseudomonas aeruginosa* or *Bacillus smithii*. (v) The conversion of aldehydes in the presence of sodium hyponitrite or *N*-hydroxy succinimide and cobalt diacetate. (vi) Solid-phase synthesis of HAs.

The synthesis of hydroxamic acids is a widely studied area of organic chemistry, with several basic approaches [5,6,7] (Figure 1). A wide range of HA derivatives have been obtained using these methods [2,3,4,5].

Recently, a simpler method for HA synthesis from *N*-substituted succinimides has been proposed [8]. This method involves treating *N*-substituted succinimides with hydroxylamine aqueous solution at room temperature for no longer than 1 h, without any additives [8,9]. This approach allows for the one-step synthesis of *N*-hydroxybutanamide derivatives and offers the potential to synthesize a variety of HA structures as biologically active compounds.

The most extensively studied biological activity of HAs is their effect on metalloenzymes involved in various pathological processes [10]. One of the earliest subjects for research on metalloenzymes as therapeutic targets was the family of matrix metalloproteinases (MMPs). MMPs are calcium- and zinc-dependent proteolytic enzymes involved in both physiological and pathological processes. A key feature of MMPs is the presence of a methionine residue in the active center and the use of zinc in the enzymatic reaction [11].

The primary function of MMPs is tissue remodeling, which is essential for various physiological processes including development, tissue homeostasis, morphogenesis, and tissue repair. However, tissue remodeling also plays a significant role in pathological conditions such as arthritis, cardiovascular diseases, neurodegenerative diseases, and developmental disorders [12,13,14,15]. MMPs are involved in the functioning of signaling systems activated at the cell surface and play a crucial role in the availability of growth factors, thereby affecting differentiation, migration, proliferation, and apoptosis [16].

MMPs are considered therapeutic targets for cancer treatment due to their involvement in the dysregulation of tissue homeostasis in malignant neoplasms, particularly their ability to remodel the extracellular matrix (ECM) and to trigger a program of cell invasion leading to increased metastasis [16,17,18].

The first class of MMP inhibitors to be extensively studied were compounds containing HA as a zinc-binding group (ZBG) [19]. Batimastat and marimastat have been widely studied in preclinical and clinical studies but have been found to have a number of side effects. Prinomastat was designed for more selective inhibition of MMP-2, -9, and -13 and MT1-MMP (MMP-14). Clinical trials have shown that, like other MMP inhibitors, it can cause side effects such as time- and dose-dependent musculoskeletal stiffness and pain [20]. Another compound, a butanoic acid derivative tanomastat, does not inhibit MMP-1, -8, or -13. Clinical trials of tanomastat have shown that patients may experience asymptomatic elevated liver enzymes and thrombocytopenia. It is worth noting that, in patients with metastatic small cell lung cancer, the disease actually worsened after treatment with tanomastat. This indicates that there is still a lack of clear understanding of the specific role of MMPs in cancer [21,22].

MMP inhibitors have been extensively tested in combination with anti-cancer drugs such as gemcitabine, cisplatin, carboplatin, or paclitaxel. While preclinical studies in laboratory animals have shown a significant reduction in tumor growth, clinical trials have not demonstrated any benefits of combination therapy [20]. The failure of MMP inhibitors in clinical trials has led to numerous efforts to develop new generations of compounds that directly or indirectly inhibit MMPs [23].

**Figure 2:**
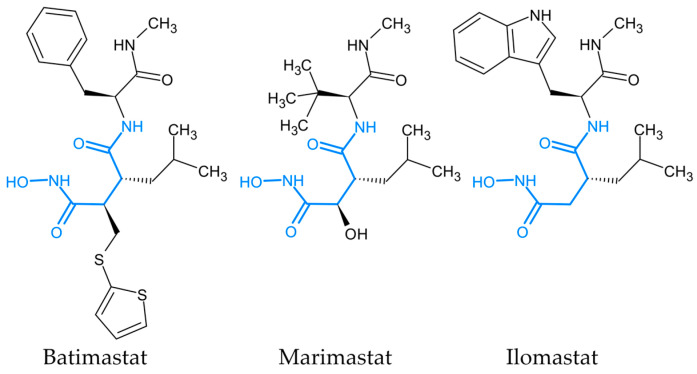
Well-known MMP inhibitors containing a *N*-hydroxybutanamide fragment (highlighted in blue).

The *N*-hydroxybutanamide fragment is commonly found in inhibitors of metalloenzymes [24,25]. In particular, MMP inhibitors such as batimastat and its analogs contain the *N*-hydroxybutanamide fragment (Figure 2), which supports the potential of our novel approach for the development of new inhibitors of metalloenzymes.

Our research focuses on synthesizing new compounds with a hydroxamic acid group and evaluating their potential biological activities. In this study, we synthesized five HA compounds with a benzene ring with different substitutions. To bring additional sources of potential hydrogen bonding, a diacylated hydrazine fragment was introduced into several compounds, as it was shown that hydrazide fragments can enhance the selectivity and antitumor activity of MMP inhibitors [26,27]. This paper presents the synthesis of five new *N*-hydroxybutanamide derivatives and the results of our studies on their MMP inhibition activity, cytotoxicity, acute toxicity, and antitumor activity.

## 2. Results

### 2.1. Synthesis of New N-Hydroxybutanamide Derivatives

**Scheme 1:**
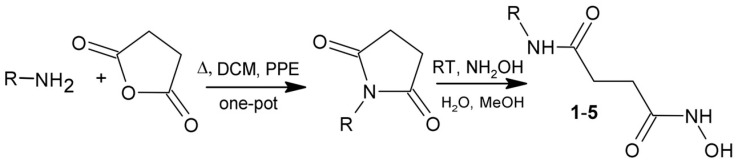
Synthesis of *N*-hydroxybutanamide derivatives.

New *N*-hydroxybutanamide derivatives were synthesized using the route depicted in Scheme 1. The initial amines or carboxylic acid hydrazides (R-NH_2_) were acylated with succinic acid anhydride, followed by an imidization reaction in the presence of polyphosphate ester (PPE) in chloroform. Both steps were carried out in a one-pot manner, as shown in the first reaction in Scheme 1. The resulting *N*-substituted succinimides were then treated with aqueous hydroxylamine in the presence of methanol (10% methanol is required to increase the purity of the final product) at room temperature, as shown in step 2 of Scheme 1.

Four benzohydrazide derivatives and one iodoaniline derivative of *N*-hydroxybutanamide were synthesized. The structures of these new compounds are shown in Table 1.

### 2.2. MMP Inhibitory Activity

Four enzymes of the MMP family were studied: MMP-2, MMP-3, MMP-9, and MMP-14. These four MMPs represent different classes within the MMP family. MMP-2 and MMP-9 belong to the gelatinase class, and in cancer, they play important roles in angiogenesis, tumor growth, invasion, and metastasis [28,29,30,31,32,33,34]. MMP-3, from the stromelysin class, is also associated with tumor progression [35,36,37] and activates MMP-9 trough partial proteolysis [38,39]. Membrane-type MMP-14, also known as MT1-MMP, is involved in the progression of various types of cancer [40,41] and is an activator of MMP-2 [42,43,44]. MMP-2 and MMP-9 are often considered targets for antitumor therapy due to their crucial roles in degrading the extracellular matrix and promoting tumor invasion and metastasis [31,32,33,34]. MMP-14 is of particular interest due to its involvement in the progression of gliomas, with elevated levels in these tumors and important functions in growth, invasion, migration, and angiogenesis. MMP-3, along with other MMPs, has been shown to affect tumor cell migration and invasion, but its improper activity has been linked mainly to osteoarthritis [37].

**Figure 3:**
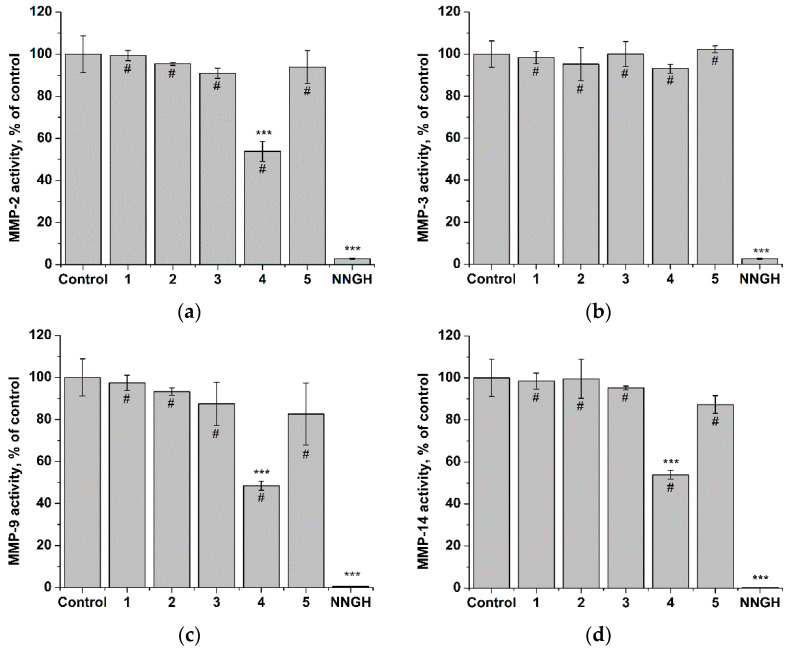
Activity of (**a**) MMP-2; (**b**) MMP-3; (**c**) MMP-9; (**d**) MMP-14 in the presence of **1**–**5** or NNGH at a concentration of 1 μM. Significant differences are shown as *** *p* < 0.001 vs. control and as # *p* < 0.001 vs. NNGH.

The results of MMP inhibition studies are presented in Figure 3 and Table 2. The nonspecific MMP inhibitor *N*-hydroxy-2-[[(4-methoxyphenyl)sulfonyl](2-methylpropyl)amino]-acetamide (NNGH) was provided as a positive control by the MMP activity assay kit manufacturer (see the Materials and Methods section for details). The diacylated hydrazine-bearing compounds showed weak inhibitory activity against MMPs. Compound **4** inhibited the activity of MMP-2, MMP-9, and MMP-14 by approximately 50%, but had no effect on MMP-3 activity (Figure 3).

**Figure 4:**
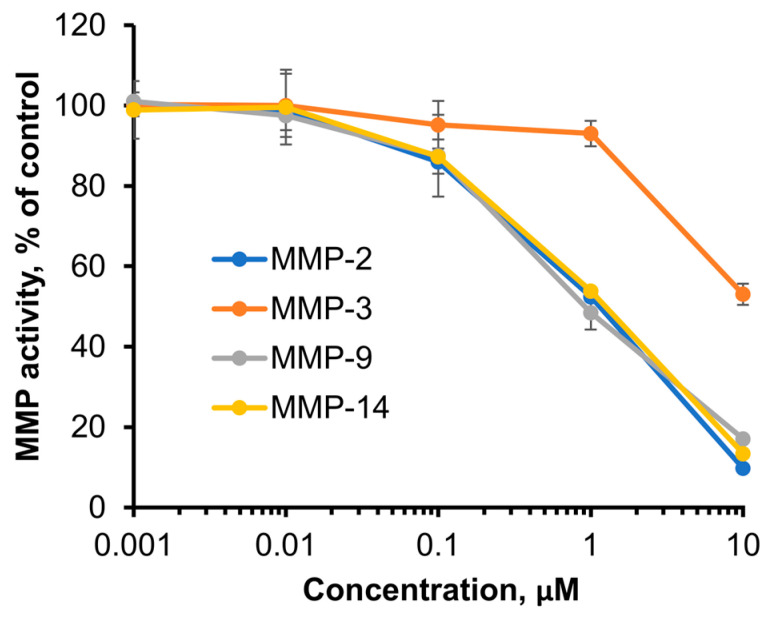
Dose–response curves for MMPs in the presence of **4**.

We further studied the dose–effect relationships for compound **4** on all four enzymes (Figure 4) and determined the IC_50_ values (Table 2). Compound **4** had a significant effect on MMP-3 activity at a relatively high concentration (approximately 10 μM), while it inhibited MMP-2, MMP-9, and MMP-14 with an IC_50_ of approximately 1 μM to 1.5 μM. Thus, the iodoaniline *N*-hydroxybutanamide derivative **4** exerts an MMP inhibitory effect that is more pronounced for MMP-2, MMP-9, and MMP-14.

### 2.3. Molecular Docking

**Figure 5:**
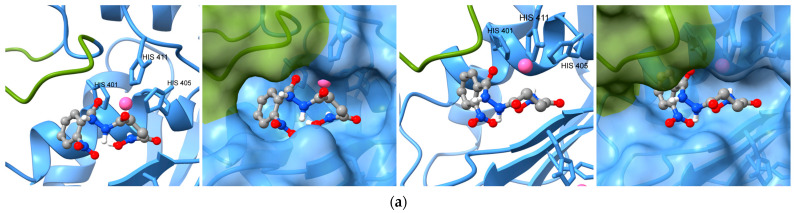

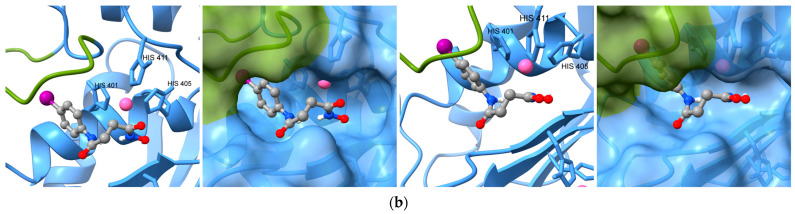
Docked poses of **1** (**a**) and **4** (**b**) at the active site of MMP-9. Complexes are represented in ball and stick style. The S1′ pocket is highlighted in green, and the zinc atom is highlighted in pink.

For the molecular docking simulation, the MMP-9 structure was used (PDB ID 1GKC [45]). For comparison, in silico simulations were conducted for compounds **1** and **4**. The results of the docking showed that the hydroxamic acid moiety of both compounds is located in close proximity to the zinc atom in the active center (Figure 5). Additionally, both molecules are directed towards the S1′ pocket of the enzyme. The binding energies for the conformations with the highest docking scores were −7.02 kcal/mol for **1** and −9.44 kcal/mol for **4**. The estimated inhibition constant values were 7.17 μM for **1** and 120.50 nM for **4**. These results are in an agreement with the MMP inhibitory activity (Table 2), as the active compound **4** demonstrated significantly lower quantitative parameters compared to the inactive compound **1**.

### 2.4. Cytotoxicity

For the cytotoxicity studies, cell lines of cancerous and non-cancerous origin were used. The cancerous cell lines included human glioblastoma cells A-172 and U-251 MG, human cervical carcinoma HeLa cells, and human hepatocellular carcinoma HepG2 cells. The non-cancerous cell lines used were fetal bone marrow stem cells FetMSC and African green monkey renal epithelium cells Vero.

**Figure 6:**
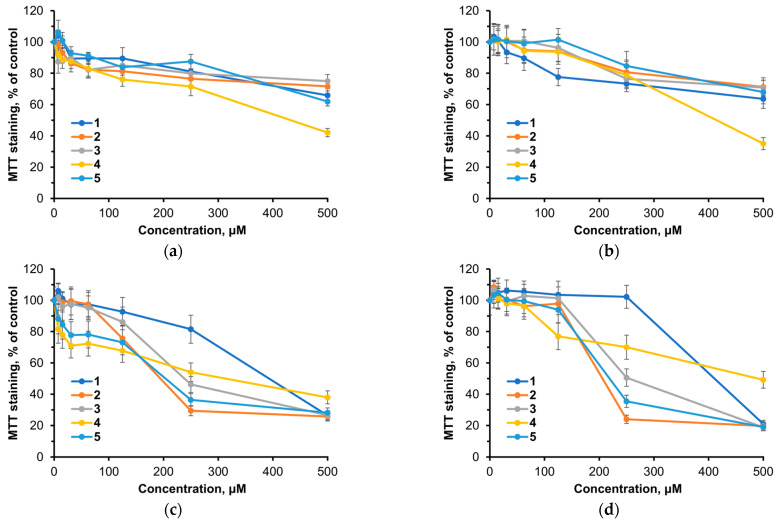

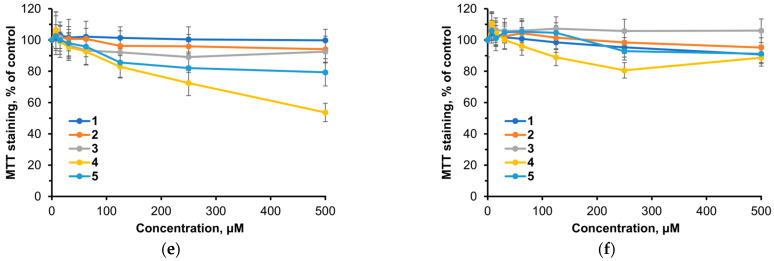
Dose–effect relationships for **1**–**5** after 72 h of exposure in cells (**a**) A-172; (**b**) U-251 MG; (**c**) HeLa; (**d**) HepG2; (**e**) FetMSC; and (**f**) Vero.

The results of the study are shown in Figure 6 and Table 3. Overall, the compounds showed low toxicity to cells, with IC_50_ values falling in the range of concentrations higher than 100 μM. According to the classification provided in [46,47], all of the compounds can be classified as little toxic to cells. However, there were differences in toxicity among the compounds towards tumor cells. While carcinoma cells HeLa and HepG2 were sensitive to all compounds, glioblastoma cells A-172 and U-251 MG showed sensitivity only to compound **4**.

The *N*-hydroxybutanamide derivatives with a benzohydrazide moiety with a *meta*- or *para*-nitro group (**2** and **3**, respectively) were found to be more toxic to carcinoma cells compared to **1**, which has an *ortho*-nitro group. Replacing the nitro group with a methoxy group at the *ortho* position (compound **5**) resulted in higher toxicity to carcinoma cells.

The non-cancerous cells FetMSC and Vero showed little to no sensitivity to all compounds. Compound **4** did exhibit some toxicity to FetMSC cells only at the highest concentration. This low or absent toxicity to non-cancerous cells is a positive indication for potential use in vivo, as it suggests less to toxicity to healthy tissues.

The low cytotoxicity of **1**–**5** is further demonstrated by comparing it to that of the clinically approved drug cisplatin. The latter was toxic to cells with IC_50_ values of approx. 10 μM.

### 2.5. Acute Toxicity In Vivo

Acute toxicity studies on laboratory animals were conducted for compound **4**, which showed MMP inhibitor activity. The compound was administered intraperitoneally at doses ranging from 200 to 1000 mg/kg. The solubility limits restricted further increases in the dose.

Compound **4** neither exhibited toxic effects nor caused the death of animals; therefore, quantitative values of lethal doses could not be established. No toxic effects or deaths were observed in animals; therefore, quantitative values for lethal doses could not be established. The animals in the experimental groups showed normal behavioral reactions, food and water intake, and respiratory rate, which did not differ from the control group. Throughout the observation period after the administration of compound **4**, no digestive disorders, changes in coat, or skin conditions were observed.

**Figure 7:**
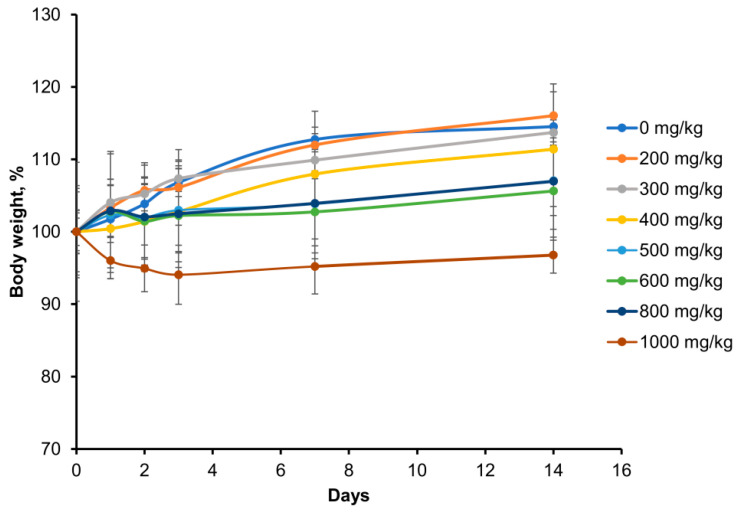
Relative body weight changes in groups of mice that received different single doses of **4**, with 100% representing the body weight at the beginning of the experiment.

In groups that received 300 mg/kg or lower doses, a stable increase in body weight was observed, while a slower weight gain was noted in the group that received 400 mg/kg. No significant changes in body weight were observed in the groups that received 600 mg/kg and 800 mg/kg during the entire observation period. In the group that received 1000 mg/kg, a slight decrease in body weight was observed within 7 days, followed by partial recovery on day 14 (Figure 7).

The morphological characteristics of the organs of all experimental animals were similar to those observed in the control group.

Thus, compound **4**, when administered intraperitoneally at doses up to 1000 mg/kg, did not exhibit toxic effects and can be classified as a slightly toxic compound [48].

### 2.6. Antitumor Activity In Vivo

**Figure 8:**
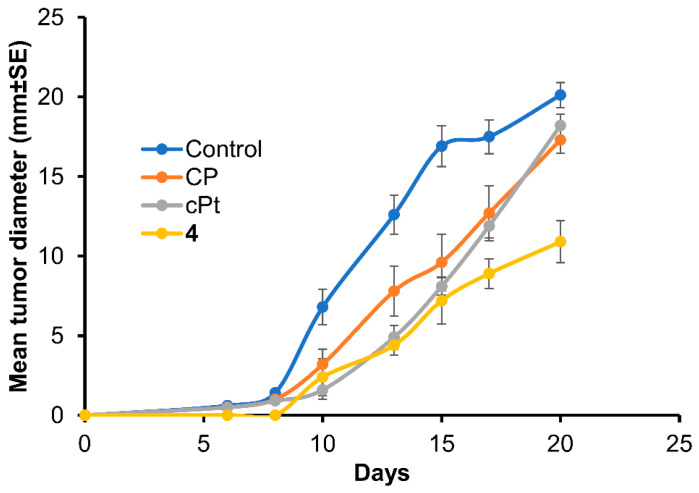
Effect of **4** on the growth of syngeneic B16 melanoma. Tumor growth was measured with a caliper, and the mean ± SE was plotted for *n* = 7 in each treatment group.

The antitumor activity of compound **4** was investigated in BDF1 mice with B16 melanoma. This model was chosen due to ease detection of metastases. The therapy consisted of intraperitoneal administration of **4** at a dose of 300 mg/kg, which did not affect the body weight of mice (see Figure 7). For comparison, cisplatin (cPt) and cyclophosphamide (CP) were used at therapeutic doses of 4 mg/kg and 100 mg/kg, respectively. These doses were determined in previous toxicity studies. The results of the study are shown in Figure 8 and Table 4.

It can be observed that the intraperitoneal injection of **4** at a dose of 300 mg/kg from days 2 to 9 after tumor transplantation resulted in the inhibition of B16 melanoma tumor growth. The most significant inhibition of tumor growth was seen on day 13 after the start of treatment. cPt and CP at the used doses showed a smaller degree of tumor growth inhibition.

All of the studied compounds exhibited strong antimetastatic activity, with **4** showing the highest effect. Our results demonstrate (Table 5) that compound **4** also reduced the number of animals with metastases in the group by 50%.

Thus, compound **4**, which has an IC_50_ of 1 to 1.5 μM for inhibiting MMPs, showed notable antitumor activity in B16 melanoma-bearing mice, while also being minimally toxic to cells in vitro and non-toxic to animals in vivo. This antitumor activity was similar to that of the approved cytotoxic drugs cisplatin and cyclophosphamide.

## 3. Discussion

New *N*-hydroxybutanamide derivatives were synthesized using a recently developed *N*-substituted succinimide ring opening approach. The results of both in vitro and in vivo studies demonstrate the effectiveness of this approach for the development of biologically active compounds.

The data from the MMP inhibition assay (Table 2) show that none of the compounds with a diacylated hydrazine fragment were able to inhibit MMPs at a concentration of 10 μM. Neither variations in the position of the nitro group nor its replacement with a methoxy group in the benzene ring resulted in apparent MMP-inhibiting activity.

Compound **4**, which contains an iodoaniline moiety, showed inhibiting activity towards MMP-2, -9, and -14 with IC_50_ values of approx. 1 to 1.5 μM (Table 2). Several compounds with carboxylic [49] and dithiol [50] ZBG have been synthesized and shown to have specificity towards MMP-2, -9, and -14. One of these compounds, carboxylic ZBG-bearing MMI-166, has been shown to have anti-invasive, anti-metastatic, and antitumor activity [51,52,53]. Thus, compound **4** may have antitumor potential as well.

It has to be noted that most MMP inhibitors developed thus far have much greater potency, with common IC_50_ values in the nanomolar range except tetracycline derivatives. The latter class of compounds inhibits MMPs at micromolar concentrations, and tetracycline derivative doxycycline is the only clinically approved MMP inhibitor [54]. Thus, we suppose that compound **4**, despite the micromolar range of IC_50_ values for MMP inhibition, represents a promising structure type.

The molecular docking study revealed that compound **4** has a higher estimated free energy of binding to MMP-9 compared to compound **1**. This suggests that compound **4** has a stronger affinity for the target enzyme, which is a key factor in inhibiting its activity. Together with the results of the MMP inhibition studies, our findings suggest that the structure of compound **4** is a promising starting point for increasing its potency and specificity. The ring opening method can be easily applied for wide structural modifications of the *N*^1^-hydroxy-*N*^4^-phenylbutanediamide scaffold.

The results of the in vitro cytotoxicity studies showed that compounds **1**–**5** were a little toxic to cells (IC_50_ higher than 100 μM), which is consistent with the literature data on many other compounds with MMP inhibiting activity.

Some MMP inhibitors can be toxic to cells. Batimastat (Figure 2), the first synthetic MMP-inhibitor studied in humans with advanced malignancies [55], has been found to inhibit the growth of pancreatic adenocarcinoma HPAC cells and rat prostate cells derived from R3327-MatLyLu Dunning tumor at a concentration of 4000 ng/mL (8.37 μM) after 24 and 48 h of exposure, respectively [56,57]. Batimastat and another peptide-based MMP inhibitor, GI254023X, have been found to be toxic to pancreatic carcinoma cells lines CD18 and MiaPaCa2 at a concentration of 5 μM after 24 h of exposure [58]. Batimastat and marimastat (Figure 2), the second MMPI evaluated in cancer patients [59], were less toxic to the human glioma U-251 cells, with a reduction in cell viability of only 20–40% at a concentration of 10 μM after 96 h of exposure [60].

On the other hand, many MMP inhibitors often demonstrate lower levels of cytotoxicity. Batimastat exhibited no toxicity towards pancreatic adenocarcinoma cells AsPC1 and Capan1 at concentrations up to 10,000 ng/mL (20.94 μM) and only mild toxicity at higher concentrations (up to 100,000 ng/mL or 209.36 μM) after 72 h of exposure [61]. Similarly, it had no apparent effect on the cell density of Shionogi hormone-responsive breast carcinoma cells at a concentration of 156 μg/mL (326.61 μM) after 96 h of exposure [62]. Marimastat, which showed toxicity to human histiocytic lymphoma cells U-937 with an IC_50_ of 10.8 μg/mL (32.59 μM), was less toxic to human hepatocellular carcinoma cells HepG2 and hamster peritoneal macrophages, with an IC_50_ higher than 200 μg/mL (603.48 μM) after 72 h of exposure [63]. After 48 h of exposure, marimastat had no effect on mouse breast carcinoma cells 4T1 and human breast carcinoma cells MDA-MB-435 at concentrations up to 50 μg/mL (150.87 μM) [64]. Prinomastat, a non-peptidomimetic MMPI with a hydroxamic acid ZBG, was found to be toxic to human malignant glioma cells U-87 at relatively high concentrations (above 100 μM) [65]. Among several compounds with different types of ZBG, only carboxylate complex PD166793 showed toxicity towards co-cultured neonatal rat ventricular fibroblasts and myocytes at concentrations above 100 μM after 24 h of exposure [66].

LY52, another non-peptidomimetic compound with a hydroxamic acid ZBG, showed low toxicity towards breast adenocarcinoma cells MDA-MB-231. At a concentration of 1000 μg/mL (2.27 mM), the compound inhibited cell viability by more than 50% only after 96 h of exposure [67]. After 48 h of exposure, LY52 was a little toxic to A549, ES-2 (human ovarian clear cell carcinoma), Hela (human cervical carcinoma), K562, MCF-7 (human breast adenocarcinoma), and MDA-MB-231 cells. The IC_50_ values for the most LY52-sensitive cells, A549 and HeLa, were approx. 78 μM and 185 μM, respectively [68].

SI-27, a peptide-based MMP inhibitor with a hydroxamic acid ZBG, was studied using several malignant glioma (U-87 MG, U-373 MG, and U-251 MG) and leukemia (U-937, HL-60, NB-4, and THP-1) cell lines. It showed low toxicity towards glioma cells, with no effect on cell viability at a concentration of 100 μg/mL (279.7 μM). The IC_50_ values for different glioma cell lines ranged from 400 to 600 μg/mL (1.1 to 1.7 mM) after 24 h of exposure [69,70]. However, SI-27 showed higher toxicity towards some leukemia cells, inhibiting the cell viability of U-937, HL-60, and NB-4 cells with an IC_50_ of 100 to 200 μM but not affecting THP-1 cells at a concentration of 200 μM [71].

The MMP-2, -9, and -14 selective inhibitor MMI-166 was a little toxic to cells derived from human cervical adenocarcinoma (designated CAC-1 cells) [53] and the hamster pancreatic cancer cell line PGHAM-1 [52]. After 72 h of exposure, the IC_50_ values were higher than 50 μg/mL (102.35 μM) for CAC-1 cells and higher than 100 μg/mL (204.7 μM) for PGHAM-1 cells.

However, there are also examples of MMP inhibitors that exhibit very high cytotoxicity. The cyclic hydroxamic acid compound pyridoxatin showed high toxicity towards 21 cell lines, with IC_50_ values ranging from 0.1 to 7.04 μg/mL (0.38 to 26.73 μM) after 48 h of exposure, and its IC_50_ for gelatinase A (MMP-2) inhibition was 15.2 μM [72]. Triazine-based compounds with a hydrazide fragment and hydroxamic acid group were highly toxic towards MDA-MB-231 and colorectal adenocarcinoma Caco-2 cells, with cytotoxicity IC_50_ values in the nanomolar range, similar to or even lower than that for MMP inhibition [73].

Thus, the cytotoxicity of MMP inhibitors can vary substantially. Usually, IC_50_ values for the inhibition of cell viability fall within the submillimolar range, but there are examples of MMP inhibitors with very high or very low cytotoxicity.

It should be noted that the high cytotoxicity of MMP inhibitors is not considered a primary endpoint of in vitro studies. In fact, since the early research, MMP inhibitors have been viewed as non-cytotoxic medications [74]. For example, batimastat has been shown to suppress MMP activity and cell invasion at concentrations that were substantially lower than those required for cytotoxic effects. It is also important that the serum levels of batimastat were substantially lower than its cytotoxic concentration [61]. Since MMP inhibitors target enzymes that are not directly involved in cell division or cell death, high cytotoxicity of candidate inhibitors may indicate off-target activity and the presence of side targets involved in key processes of cell physiology rather than the ECM rearrangement.

Compound **4** exhibits mostly slight or absent cytotoxicity, indicating a low likelihood of off-target effects. Our findings align with previous studies on MMP inhibitors, suggesting that compounds of this structure may have potential as low-toxic anticancer agents. However, it is important to note that compounds **1**–**5** may have activity against other metalloenzymes, which requires further investigation.

Compound **4**, which showed MMP-inhibiting ability, was studied in vivo. The compound was found to exert no acute toxicity to mice at doses up to 1000 mg/kg. Previous research on various HA derivatives demonstrated low acute toxicity, with LD_50_ dose ranging from 500 to 1250 mg/kg [75,76,77]. Unlike these HAs, compound **4** did not cause any deaths in animals and did not affect their behavior or the morphological characteristics of their organs at any concentration. Additionally, at doses up to 300 mg/kg, it did not impact weight gain. These results indicate that compound **4** has low toxicity despite its MMP-inhibiting activity, which might be associated with side effects [78].

In experimental chemotherapy studies, compound **4** showed significant inhibition of tumor growth and metastasis in B16 melanoma-bearing animals, surpassing the efficacy of cisplatin and cyclophosphamide (Figure 5 and Figure 6, and Table 4 and Table 5).

Our findings demonstrating the effectiveness of MMP inhibitor monotherapy are consistent with that from previous research. For example, batimastat has been shown to inhibit tumor growth in mice with B16-BL6 melanoma [79]. Similarly, prinomastat (AG3340) has been found to inhibit the growth of human MV522 lung cancer cells in nude mice [80] and Lewis lung carcinoma [81]. Another study showed that the thiirane-based compound ND-322 was able to inhibited the growth of WM266-4 melanoma cells in nude mice [82]. The heterocyclic bidentate-based MMP inhibitor RO 28-2653 was found to inhibit the growth of the hormone-sensitive prostate cancer G subline of Dunning tumor in rats [83].

Given the low acute toxicity and MMP inhibitory activity of iodoaniline-bearing *N*-hydroxybutanamide, further research should be conducted to investigate its chronic toxicity and antitumor efficacy. Furthermore, the development of new structural variants of *N*^1^-hydroxy-*N*^4^-phenylbutanediamide derivatives is also to be performed. Our future work will focus on *N*-hydroxybutanamides with different aniline derivatives in order to study structure–activity relationships and to improve the MMP inhibition activity of these compounds.

## 4. Materials and Methods

### 4.1. Chemistry

All compounds were synthesized using a recently described novel approach [8].

Elemental analysis was performed using a CHNS/O analyzer Vario EL cube (Elementar, Langenselbold, Germany). IR spectra were obtained using an Alpha FT-IR spectrometer (Bruker, Billerica, MA, USA), with all samples being directly analyzed without dilution in KBr. ^1^H and ^13^C NMR spectra were acquired on a DRX 500 spectrometer (Bruker, Billerica, MA, USA) in DMSO-*d*_6_ with TMS serving as the internal standard. Mass spectra were recorded on a MAT INCOS 50 (Thermo Finnigan, San Jose, CA, USA) mass spectrometer with direct sample injection (EI ionization, 70 eV).

*N*-hydroxy-4-[2-(2-nitrobenzoyl)hydrazinyl]-4-oxobutanamide (**1**). Colorless crystals (yield 78.2%). Found, %: C 44.72; H 3.98; N 18.89; O 32.54. C_11_H_12_N_4_O_6_. Calculated, %: C 44.60; H 4.08; N 18.91; O 32.41. Mass spectrum, *m*/*z* (Irel, %): 265 (0.3), 264 (2.9), 263 (1.5), 182 (4.5), 181 (2.0), 161 (1.5), 151 (8.5), 150 (100), 135 (5.5), 134 (39.3), 121 (45.2), 116 (10.4), 115 (25.7), 105 (7.3), 104 (51.0), 93 (12.9), 92 (8.8), 87 (7.5), 78 (8.8), 76 (52.8), 65 (6.3), 55 (10.3), 51 (16.0), 50 (17.7), 42 (6.85), 30 (10.0), 28 (9.0). IR spectrum, ν, cm^−1^: 3231, 3197, 1623, 1594, 1572, 1545, 1525, 1494, 1460, 1441, 1361, 1312, 1283, 1227, 1166, 1155, 1146, 1077, 1044, 986, 962, 879, 852, 788, 771, 736, 701, 665, 636, 593, 547, 477, 419, 395. ^1^H NMR (DMSO-*d*_6_), ppm (*J*, Hz): 2.24 (2H, t, *J* = 7.6, -CH_2_-), 2.44 (2H, t, *J* = 7.5, -CH_2_-), 7.64–7.68 (1H, m, Ar-H); 7.72–7.75 (1H, m, Ar-H), 7.82–7.86 (1H, m, Ar-H), 8.06–8.09 (1H, m, Ar-H). 8.72 (1H, s, N-OH), 9.75–10.55 (3H, broad signal, C(O)N-H). ^13^C NMR spectrum (DMSO-*d*_6_), δ, ppm: 27.33; 28.43; 124.16; 129.39; 130.25; 131.28; 133.60; 147.06; 164.17; 168.01; 170.17.

*N*-hydroxy-4-[2-(3-nitrobenzoyl)hydrazinyl]-4-oxobutanamide (**2**). Colorless crystals (yield 81.8%). Found, %: C 44.65; H 4.10; N 18.98; O 32.49. C_11_H_12_N_4_O_6_. Calculated, %: C 44.60; H 4.08; N 18.91; O 32.41.Mass spectrum, *m*/*z* (*I*_rel_, %): 263 (4.7), 151 (6.9), 150 (100), 134 (1.2), 105 (2.4), 104 (40.7), 92 (4.4), 77 (3.6), 76 (48.8), 63 (4.5), 57 (4.8), 56 (5.9), 55 (13.3), 50 (23.6), 46 (6.1), 43 (6.2), 42 (16.6), 31 (26.5), 30 (41.8), 29 (23.1), 28 (45.6), 27 (26.8), 26 (17.1), 16 (32.4). IR spectrum, ν, cm^−1^: 3283, 3081, 1692, 1662, 1644, 1619, 1582, 1533, 1478, 1440, 1354, 1327, 1305, 1275, 1255, 1152, 1101, 1066, 1025, 1002, 968, 929, 917, 819, 763, 733, 712, 670, 619, 546, 508, 473, 401. ^1^H NMR (DMSO-*d*_6_), ppm (*J*, Hz): 2.26 (2H, t, *J* = 7.6, -CH_2_-), 2.47 (2H, t, *J* = 7.6, -CH2-), 7.79–7.85 (1H, m, Ar-H); 8.27–8.31 (1H, m, Ar-H), 8.43 (1H, dd, *J*_1_ = 8.2, *J*_2_ = 2.1, Ar-H) 8.69 (1H, s, Ar-H), 8.71 (1H, s, N-OH), 10.07 (1H, s, C(O)NH), 10.43 (1H, s, C(O)NH), 10.73 (1H, s, C(O)NH). ^13^C NMR spectrum (DMSO-*d*_6_), δ, ppm: 27.40; 28.55; 122.09; 126.34; 130.29; 133.68; 133.75; 147.60; 163.35; 167.98; 170.51.

*N*-hydroxy-4-[2-(4-nitrobenzoyl)hydrazinyl]-4-oxobutanamide (**3**). Colorless crystals (yield 79.8%). Found, %: C 44.58; H 4.12; N 18.96; O 32.50. C_11_H_12_N_4_O_6_. Calculated, %: C 44.60; H 4.08; N 18.91; O 32.41.Mass spectrum, *m*/*z* (*I*_rel_, %): 263 (4.4), 244 (1.2), 158 (1.2), 150 (100), 120 (3.5), 92 (1.9), 76 (15.8), 75 (1.6), 55 (3.0), 50 (4.4), 47 (8.4), 46 (7.1), 42 (8.5), 33 (5.6), 31 (31.7), 28 (49.7), 27 (26.9), 26 (3.7). IR spectrum, ν, cm^−1^: 3573, 3336, 3223, 3010, 2866, 1692, 1649, 1598, 1538, 1504, 1425, 1352, 1325, 1285, 1228, 1158, 1091, 1068, 1037, 995, 974, 905, 869, 854, 817, 785, 699, 657, 596, 549, 516, 467, 425, 384. ^1^H NMR (DMSO-*d*_6_), ppm (*J*, Hz): 2.26 (2H, t, *J* = 7.6, -CH_2_-), 2.46 (2H, t, *J* = 7.5, -CH_2_-), 8.08 (2H, d, *J* = 8.8, Ar-H), 8.32–8.36 (2H, m, Ar-H), 8.70 (1H, s, N-OH), 10.05 (1H, s, C(O)NH), 10.30–10.80 (2H, broad signal, C(O)NH). ^13^C NMR spectrum (DMSO-*d*_6_), δ, ppm: 27.38; 28.55; 123.59; 123.72; 128.87; 138.03; 149.25; 163.82; 167.98; 170.49.

*N*^1^-hydroxy-*N*^4^-(4-iodophenyl)butanediamide (**4**). Colorless crystals (yield 85.2%). Found, %: C 36.01 H 3.40; N 8.44; O 14.49. C_10_H_11_IN_2_O_4_. Calculated, %: C 35.95; H 3.32; I 37.98; N 8.38; O 14.37. Mass spectrum, *m*/*z* (*I*_rel_, %): 335 (1), 334 [M]^+^ (7.9), 302 (12.0), 246 (3.9), 245 (7.7), 220 (6.0), 219 (100), 218 (23.7), 203 (4.3), 191 (5.3), 176 (1.4), 175 (8.1), 174 (1.2), 161 (1.7), 150 (2.5), 146 (3.2), 128 (2.8), 127 (24.3), 119 (9.5), 118 (6.0), 116 (15.6), 105 (1.6), 104 (4.3), 93 (7.8), 92 (26.1), 91 (33.0), 90 (17.9), 88 (6.5), 87 (1.9), 76 (16.9), 75 (7.2), 74 (7.2), 70 (9.4), 65 (24.5), 64 (29.9), 63 (40.1), 62 (12.8), 60 (16.2), 56 (23.9), 55 (58.0), 52 (9.6), 50 (15.1), 44 (18.7), 43 (12.4), 42 (33.9), 39 (15.3), 38 (12.6), 33 (22.4), 32 (32.3), 30 (10.3), 29 (44.2), 28 (100), 27 (67.5), 26 (22.5), 18 (3.9), 17 (4.7), 16 (5.6), 15 (4.2). IR spectrum, ν, cm^−1^: 3252, 2717, 1654, 1618, 1584, 1527, 1482, 1431, 1419, 1386, 1349, 1302, 1287, 1240, 1189, 1165, 1087, 1059, 1043, 1005, 968, 856, 846, 814, 781, 746, 706, 662, 601, 549, 501, 467, 399, 377. ^1^H NMR (DMSO-*d*_6_), ppm (*J*, Hz): 2.28 (2H, t, *J* = 7.2, -CH_2_-), 2.55 (2H, t, *J* = 7.2, -CH_2_-), 7.42 (2H, d, *J* = 8.7, Ar-H), 7.61 (2H, d, *J* = 8.7, Ar-H), 8.69 (1H, s, N-OH), 10.04 (1H, s, C(O)NH), 10.41 (1H, s, C(O)NH). ^13^C NMR spectrum (DMSO-*d*_6_), δ, ppm: 27.15; 31.37; 86.11; 120.99; 137.18; 138.99; 168.17; 170.30.

*N*-hydroxy-4-[2-(2-methoxybenzoyl)hydrazinyl]-4-oxobutanamide (**5**). Colorless crystals (yield 75.6%). Found, %: C 51.31; H 5.41; N 15.02; O 28.51. C_12_H_15_N_3_O_4_. Calculated, %: C 51.24 H 5.38; N 14.94; O 28.44.Mass spectrum, *m*/*z* (*I*_rel_, %): 248 (3.0), 166 (2.2), 136 (8.5), 135 (100), 121 (1.3), 120 (2.8), 105 (2.4), 92 (23.7), 79 (5.5), 78 (4.43), 77 (50.1), 74 (2.8), 65 (4.7), 64 (13.5), 63 (13.3), 57 (4.6), 56 (7.8), 55 (17.3), 51 (11.3), 50 (7.4), 44 (8.9), 42 (13.2), 39 (6.4), 38 (5.6), 33 (2.7), 32 (4.1), 31 (11.4), 29 (17.9), 28 (47.9), 27 (24.7), 26 (11.4), 18 (7.0), 17 (4.7), 16 (7.9), 15 (20.7). IR spectrum, ν, cm^−1^: 3272, 3189, 3026, 1663, 1613, 1596, 1561, 1536, 1493, 1477, 1417, 1382, 1289, 1264, 1239, 1218, 1178, 1114, 1077, 1050, 988, 915, 855, 763, 677, 651, 609, 580, 531, 482, 404. ^1^H NMR (DMSO-*d*_6_), ppm (*J*, Hz): 2.26 (2H, t, *J* =7.5, -CH_2_-), 2.46 (2H, t, *J* = 7.5, -CH_2_-), 3.91 (3H, s, -CH_3_), 7.08 (1H, t, *J* = 7.4, Ar-H), 7.18 (1H, d, *J* = 8.3, Ar-H), 7.50–7.56 (1H, m, Ar-H), 7.76 (1H, d, *J* = 7.6, Ar-H), 8.71 (1H, s, N-OH), 9.93 (1H, s, C(O)NH), 10.09–10.30 (1H, broad signal, C(O)NH), 10.43 (1H, s, C(O)NH). ^13^C NMR spectrum (DMSO-*d*_6_), δ, ppm: 26.19; 26.54.70; 27.20; 27.42; 28.47; 55.66; 55.83; 111.87; 112.00; 120.36; 120.47; 121.11; 129.79; 130.34; 132.50; 132.69; 156.90; 163.35; 168.06; 169.47.

### 4.2. Biological Studies

**Determination of MMP activity**. A fluorescently labeled substrate probe containing a fluorescent label and a fluorescence quencher, was utilized for this study. Upon cleavage, the fluorescent label was released from the substrate and escaped quenching; inhibition of the enzyme resulted in a decrease in fluorescence. Enzyme activity was assessed using the MMP Inhibitor Profiling Kit (Product # BML-AK308, New York, NY, USA) according to the manufacturer’s recommendations. The nonspecific inhibitor NNGH was used as a positive control. The fluorescence of the fluorogenic substrate OmniMMP^TM^ RED was measured at wavelengths Ex/Em = 545/576 nm using a Spark 10M multimode plate reader (Tecan, Zurich, Switzerland). Reaction rates were expressed in relative fluorescence units (RFUs) per minute. The activity of MMPs in the presence of the studied compounds was determined as follows:

Activity, % = (V_inh_/V_cont_) × 100, (1)

where V is the initial reaction rate (RFUs/min) in the presence (V_inh_) and absence (V_cont_) of the studied compounds.

All tested compounds were dissolved in DMSO. The final concentration of DMSO in all samples was 0.1%. Control samples were treated with 0.1% DMSO.

**Molecular Docking.** The protein structure of MMP-9 (PDB ID 1GKC [45]) was downloaded from www.rcsb.org (accessed on 6 November 2023). Using ChimeraX 1.6.1 [84], the protein molecule was prepared by removing the co-crystalized ligand, water molecules, and chain B. The charges of ions were assigned using the AM1-BCC method. A preliminary re-docking of the co-crystalized ligand resulted in acceptable reference RMSD values for the lowest energy ligand poses (1.1 to 1.6). The geometry of the compounds was optimized using Avogadro 1.2.0 [85] with an MMFF94 force field. Docking was performed using AutoDock 4.2.6 software within the AutoDockTools4 software package [86]. To generate 3D affinity grid fields, AutoGrid4 was used with a grid map of 40 × 40 × 40 and a spacing of 0.375 Å. The grid box was centered at coordinates of 65.714, 30.223, and 117.65. All rotatable bonds in the compounds were allowed to rotate, and the protein molecule was set rigid. After simulations, the conformations of the compounds with the lowest binding energy were chosen for visualization of the protein–ligand complexes using ChimeraX 1.6.1 software.

**Cell culture**. A-172 (human glioblastoma cells), HepG2 (human hepatocellular carcinoma cells), M-HeLa (human cervical adenocarcinoma cells, M subclone), Vero (African green monkey kidney epithelial cells), and FetMSC (human embryonic mesenchymal stem cells) were purchased from the Russian Collection of Cell Cultures of Vertebrates (Institute of Cytology RAS, St. Petersburg, Russia). The U-251 MG cell line (human glioblastoma-astrocytoma) was purchased from the European Collection of Authenticated Cell Cultures (Salisbury, UK). All cells were grown at 37 °C in an atmosphere of 5% CO_2_. A-172 and Vero cells were cultured in DMEM medium (PanEco, Moscow, Russia). HepG2, HeLa, and U-251 MG cells were cultured in EMEM medium (PanEco). FetMSC cells were cultured in F12/DMEM medium (PanEco). All incubation media were supplemented with 10% fetal calf serum (BioWest, Nuaillé, France), 50 U/mL penicillin, and 50 mg/mL streptomycin (PanEco). Additionally, media for HeLa, HepG2, and U-251 MG cells were supplemented with 2 mM glutamine (PanEco) and 1% essential amino acids (PanEco), media for A-172 and Vero cells were supplemented with 4 mM glutamine, and the medium for FetMSC cells was supplemented with 3 mM glutamine. The medium for U-251 MG cells was additionally supplemented with 1 mM sodium pyruvate (PanEco).

**Cytotoxicity studies**. The cytotoxicity of the compounds was assessed using the MTT test. Cells were seeded into 96-well plates at varying concentrations of 5 × 10^4^ cells/mL (A-172, Vero and HeLa), 7 × 10^4^ cells/mL (HepG2 and U-251 MG), and 10 × 10^4^ cells/mL (FetMSC). The studied compounds were added to the incubation medium 24 h after seeding at concentrations ranging from 7.8 μM to 500 μM. All compounds were dissolved in DMSO and diluted with the incubation medium to their final concentrations just before use. The incubation medium in wells was aspirated and replaced with medium containing the studied compounds. The final concentration of DMSO in all samples was 0.1%. Control samples were treated with 0.1% DMSO. After 72 h of exposure, 3-(4,5-dimethylthiazol-2-yl)-2,5-diphenyl-2H-tetrazolium bromide (MTT) (Dia-M, Moscow, Russia) was added to the incubation medium at a concentration of 0.5 mg/mL. The resulting MTT-formazan crystals were dissolved in DMSO. The optical density was measured at 570 nm with a background wavelength of 620 nm using a Spark 10M plate reader. IC_50_ values were determined using a median effect analysis from the dose–effect relationships [87].

**Acute toxicity**. Experiments involving animals were conducted at the Unique Scientific Unit “Nursery and Vivarium of the FRC PCP MC RAS” in accordance with the rules established by the Commission on Bioethics of the FRC PCP MC RAS (protocol No. 7/22 dated 20 June 2022) and the European Convention for the Protection of Vertebrate Animals for experimental and scientific purposes. Clinically healthy BDF_1_ hybrid males weighing 20–22 g were utilized. The mice were housed in a conventional vivarium with a 12 h light regime and provided with free access to water and food. Each experimental group consisted of six animals. Compound **4** was dissolved in DMSO and then diluted with 0.9% NaCl to reduce the DMSO concentration to 10%. The solution was administered intraperitoneally at single doses ranging from 200 mg/kg to 1000 mg/kg. The control group received 10% DMSO in 0.9% NaCl intraperitoneally. The animals were monitored daily for 14 days, with attention given to their overall condition, activity, and appetite. At the end of the experiment, all animals were euthanized by inhalation of ether, followed by a pathoanatomical autopsy.

**Antitumor activity in vivo**. The B16 mouse melanoma was used as a tumor model. The B16 melanoma tumor strain was induced in male BDF_1_ mice by subcutaneous inoculation of 0.2–0.5 mL of a suspension of tumor tissue in medium 199 at intervals of 12–16 days. For the experiments, BDF_1_ mice were inoculated with 0.3 mL of a suspension of tumor tissue diluted in a 0.9% NaCl solution at a ratio of 1:5 subcutaneously on the right side.

Compound **4** was dissolved in a 0.9% NaCl solution with 1% DMSO. Cisplatin and cyclophosphamide were purchased from Teva (Tel Aviv-Yafo, Israel) and Veropharm (Moscow, Russia), respectively. All compounds were dissolved on the day of administration and given intraperitoneally at doses of 300 mg/kg (compound **4**), 4 mg/kg (cisplatin), and 100 mg/kg (cyclophosphamide). Control animals were injected intraperitoneally with 1% DMSO in saline (0.9% NaCl) in the same volumes as the test compounds.

Tumor growth was monitored on days 0, 6, 8, 10, 13, 15, 17, and 20 by measuring the tumor size with a digital caliper. The mean tumor diameter was calculated using the formula:

D = 3√a × b × c,(2)

where a, b, and c are mutually perpendicular tumor diameters.

Tumor growth inhibition was calculated using the following formula:

TGI% = [(D_cont_. − D_exp_.)/D_cont_.] × 100%, (3)

where D_exp_. and D_cont_. are the tumor diameters in the experimental and control groups, respectively.

The percentage of inhibition of metastases was calculated using the following formula:

IM% = [(MNM_cont_. – MNM_exp_.)/MNM_cont_.] × 100%, (4)

where MNM_exp_. And MNM_cont_. are the mean number of metastases in the experimental and control groups, respectively.

**Statistical data.** The experiments were conducted in at least five replicates. The data from three independent experiments are presented as X ± SD (mean ± standard deviation). The significance of differences between groups was determined using a one-way analysis of variance (ANOVA). *p* values < 0.05 were considered statistically significant. Statistical data processing was performed using GraphPad Prism 5.0 software.

## 5. Conclusions

New *N*-hydroxybutanamide derivatives were synthesized using the *N*-substituted succinimide ring opening approach, and their biological activity was characterized. One of the new compounds, the iodoaniline *N*-hydroxybutanamide derivative, inhibited MMP-2, MMP-9, and MMP-14 with an IC_50_ concentration of approx. 1–1.5 μM, with a lesser effect on MMP-3. In silico simulations demonstrated that **4** had a lower binding energy compared to the inactive complex **1**. All *N*-hydroxybutanamide derivatives showed low or no toxicity towards several cancerous cell lines and were on a little toxic to non-cancerous cells. The iodoaniline *N*-hydroxybutanamide derivative demonstrated significant antitumor and antimetastatic effects in animals with implanted B16 melanoma. Thus, the new approach to synthesizing *N*-hydroxybutanamide derivatives shows promise for developing inhibitors of metalloenzymes. In particular, the *N*^1^-hydroxy-*N*^4^-phenylbutanediamide structure type has potential for developing new inhibitors of metalloproteases with promising antitumor properties.

## Figures and Tables

**Table 1:** Structures of new *N*-hydroxybutanamide derivatives.

Compound	Structure
**1**	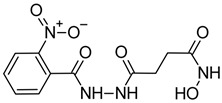
**2**	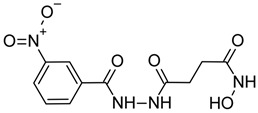
**3**	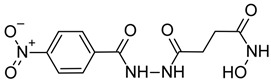
**4**	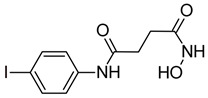
**5**	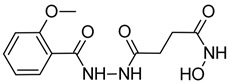

**Table 2:** MMP-inhibiting activity IC_50_ values (μM) for **1**–**5**.

Compound	MMP-2	MMP-3	MMP-9	MMP-14
**1**	>10	>10	>10	>10
**2**	>10	>10	>10	>10
**3**	>10	>10	>10	>10
**4**	1.26 ± 0.01	>10	1.06 ± 0.01	1.49 ± 0.01
**5**	>10	>10	>10	>10

**Table 3:** IC_50_ values (μM) for **1**–**5** after 72 h of exposure.

Compound	A-172	U-251 MG	HeLa	HepG2	FetMSC	Vero
**1**	>500	>500	376.7 ± 25.2	392.7 ± 26.2	>500	>500
**2**	>500	>500	184.9 ± 15.3	213.0 ± 15.3	>500	>500
**3**	>500	>500	237.0 ± 14.2	251.8 ± 15.8	>500	>500
**4**	418.3 ± 17.4	400.6 ± 21.1	296.8 ± 15.7	487.5 ± 23.9	>500	>500
**5**	>500	>500	194.5 ± 13.4	220.9 ± 12.6	>500	>500
**Cisplatin**	8.46 ± 0.92	- *	11.74 ± 2.41	13.21 ± 1.62	- *	10.74 ± 1.45

* Not determined.

**Table 4:** Tumor growth inhibition of B16 melanoma after treatment with CP, cPt, and compound **4**.

Compound	Dose, mg/kg	Administration Regimen, Day	Tumor Growth Inhibition, %				
			Day 10	Day 13	Day 15	Day 17	Day 20
CP	100	2; 7	52.94	38.10	43.20	27.43	14.02
cPt	4	2–9	76.47	61.11	52.07	32.00	9.54
**4**	300	2–9	34.5	61.5	50.4	44.6	42.3

**Table 5:** Effects of CP, cPt, and compound **4** on the processes of metastasis of experimental B16 melanoma in the lungs of BDF_1_ hybrid mice.

Compound	Dose, mg/kg	Administration Regimen, Day	Number of Animals in the Group	Number of Animals without Metastases	Metastasis Rate, %	Mean Number of Metastases per Mouse	% Inhibition of Metastases
Control	-	2–9	6	0	100	11.7 ± 3.4	-
CP	100	2; 7	6	0	100	7.1 ± 2.2	39.3
cPt	4	2–9	6	1	83.3	3.3 ± 2.4 *	71.9
**4**	300	2–9	6	3	50	1.3 ± 0.9 **	88.6

Significant differences are shown as * *p* < 0.05, and ** *p* < 0.01 vs. control.

## Data Availability

The authors confirm that the data supporting the findings of this study are available within the article and its Supplementary Materials Figures S1–S20.

## Supplementary Materials

The supporting information can be downloaded at https://www.mdpi.com/article/10.3390/ijms242216360/s1.
