# Supplementary material for: New Derivatives of N-Hydroxybutanamide: Preparation, MMP Inhibition, Cytotoxicity, and Antitumor Activity
Source: Int J Mol Sci. 2023 Nov 15;24(22):16360. doi: 10.3390/ijms242216360 (PMC10671431; doi:10.3390/ijms242216360)
Supplement: Supplementary file 1 [file ijms-24-16360-s001.zip › ijms-2647823-supplementary.pdf]

New derivatives of *N*-hydroxybutanamide: preparation, MMP inhibition, cytotoxicity and antitumor activity

Anastasia Balakina, Svyatoslav Gadomsky, Tatyana Kokovina, Tatyana Sashenkova, Denis Mishchenko, and Alexei Terentiev

N-hydroxy-4-[2-(2-nitrobenzoyl)hydrazinyl]-4-oxobutanamide (1)

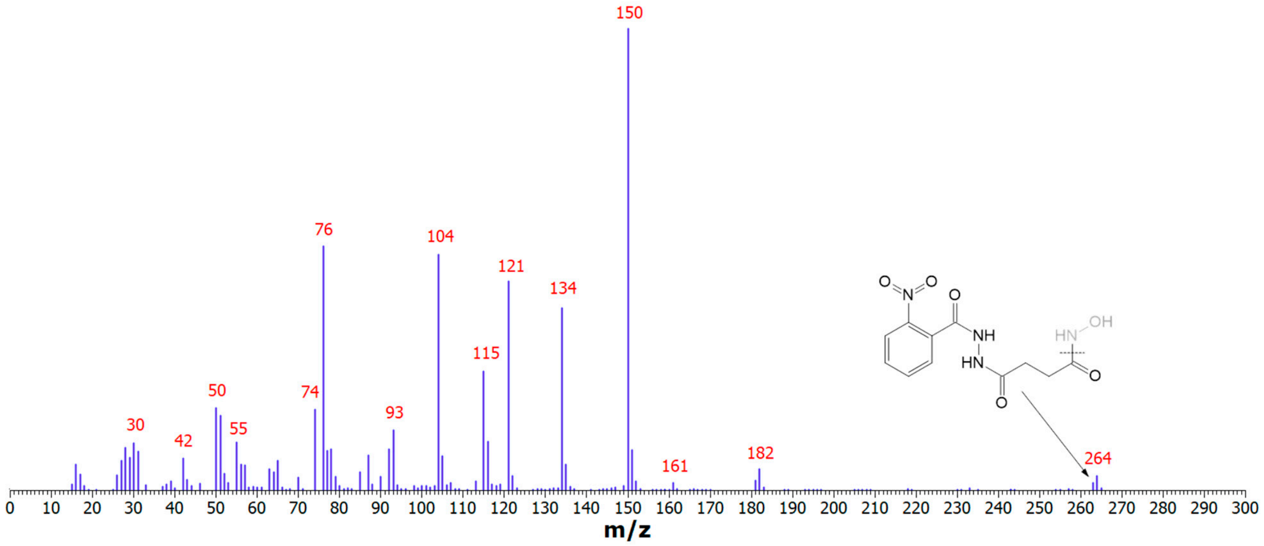

Figure S1 – Mass spectrum view of **1**

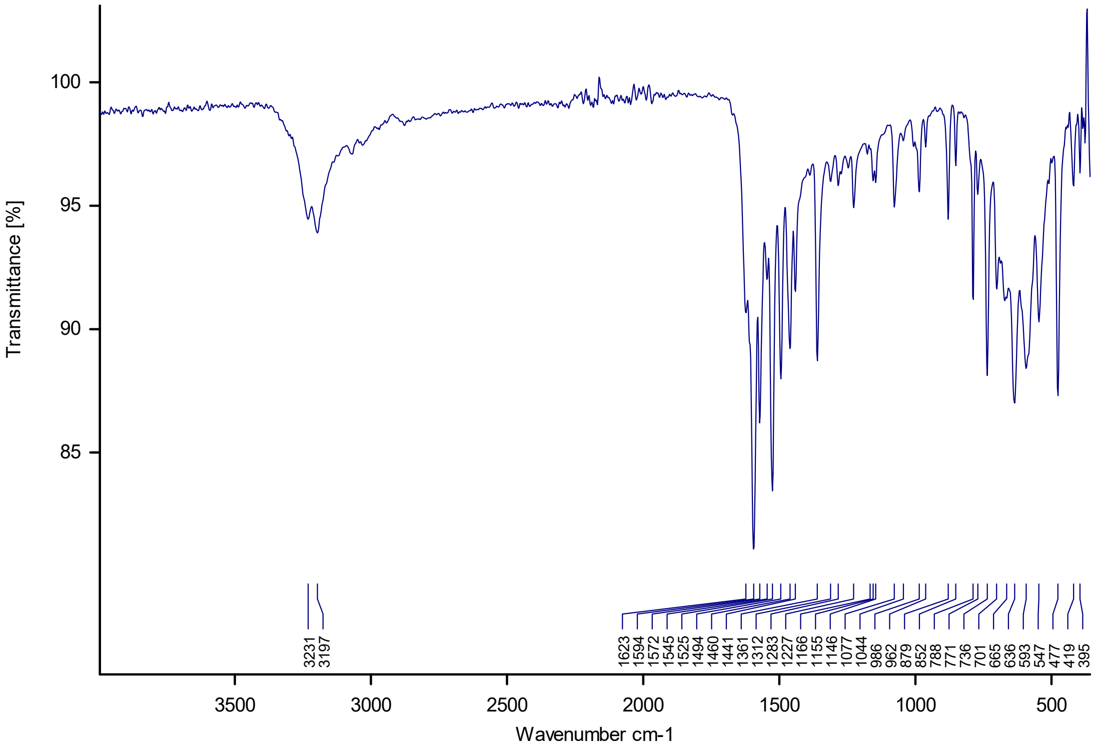

Figure S2 – IR spectrum view of **1**

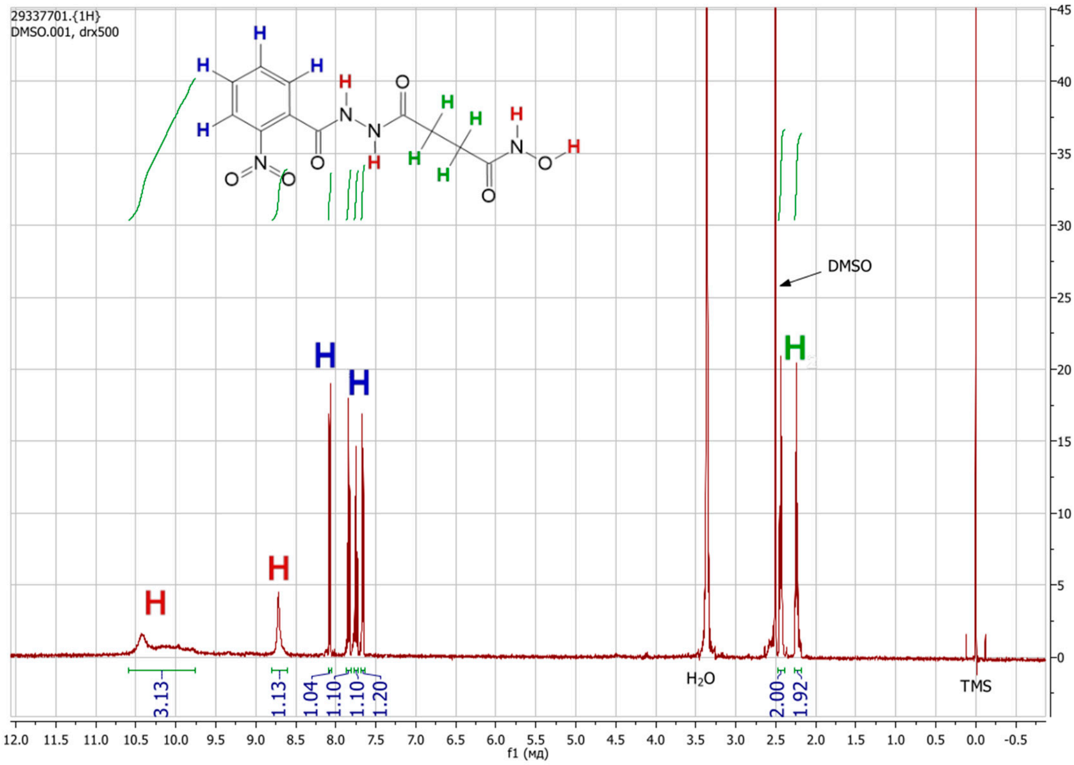

Figure S3 – ^{1}H NMR spectrum view of **1** in DMSO

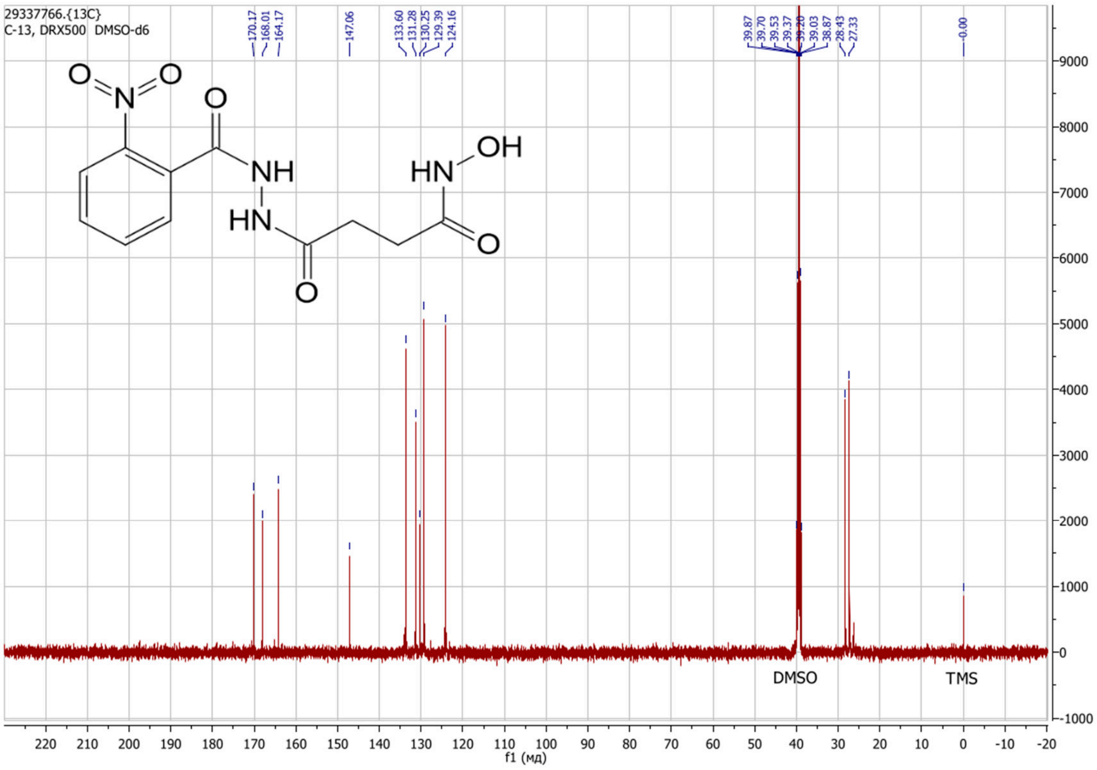

Figure S4 – ^{13}C NMR spectrum view of **1** in DMSO

_N_-hydroxy-4-[2-(3-nitrobenzoyl)hydrazinyl]-4-oxobutanamide (2)

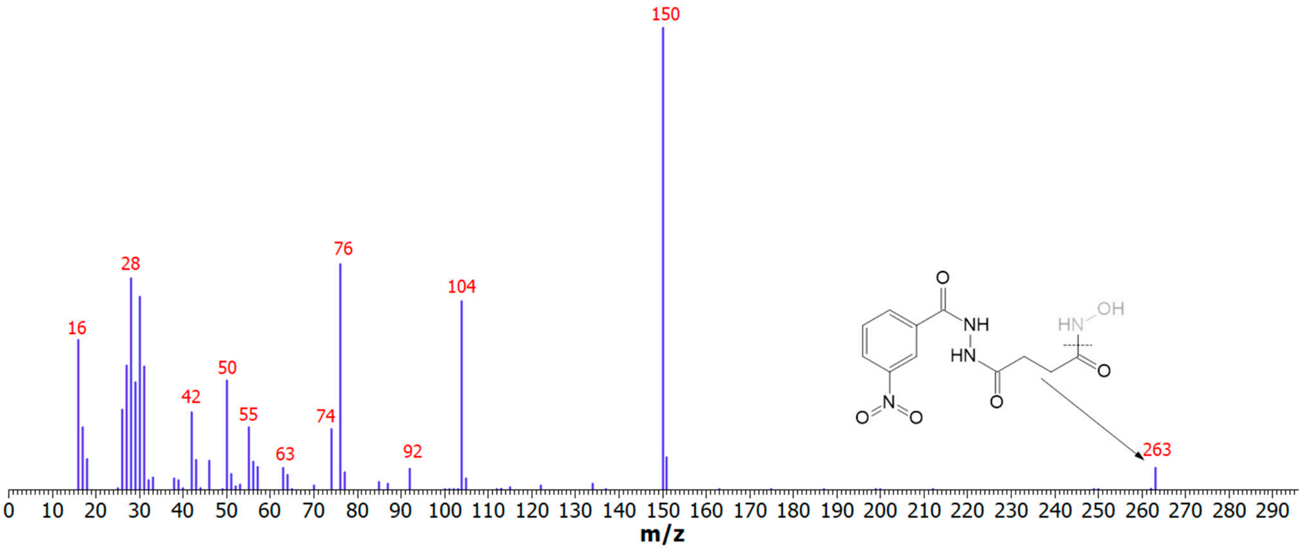

Figure S5 – Mass spectrum view of **2**

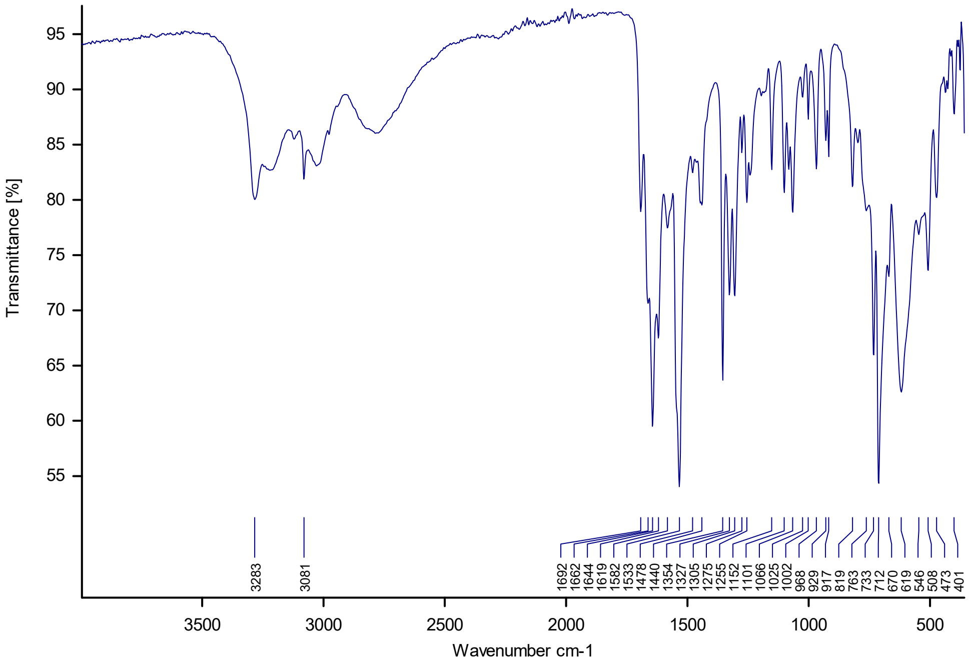

Figure S6 – IR spectrum view of **2**

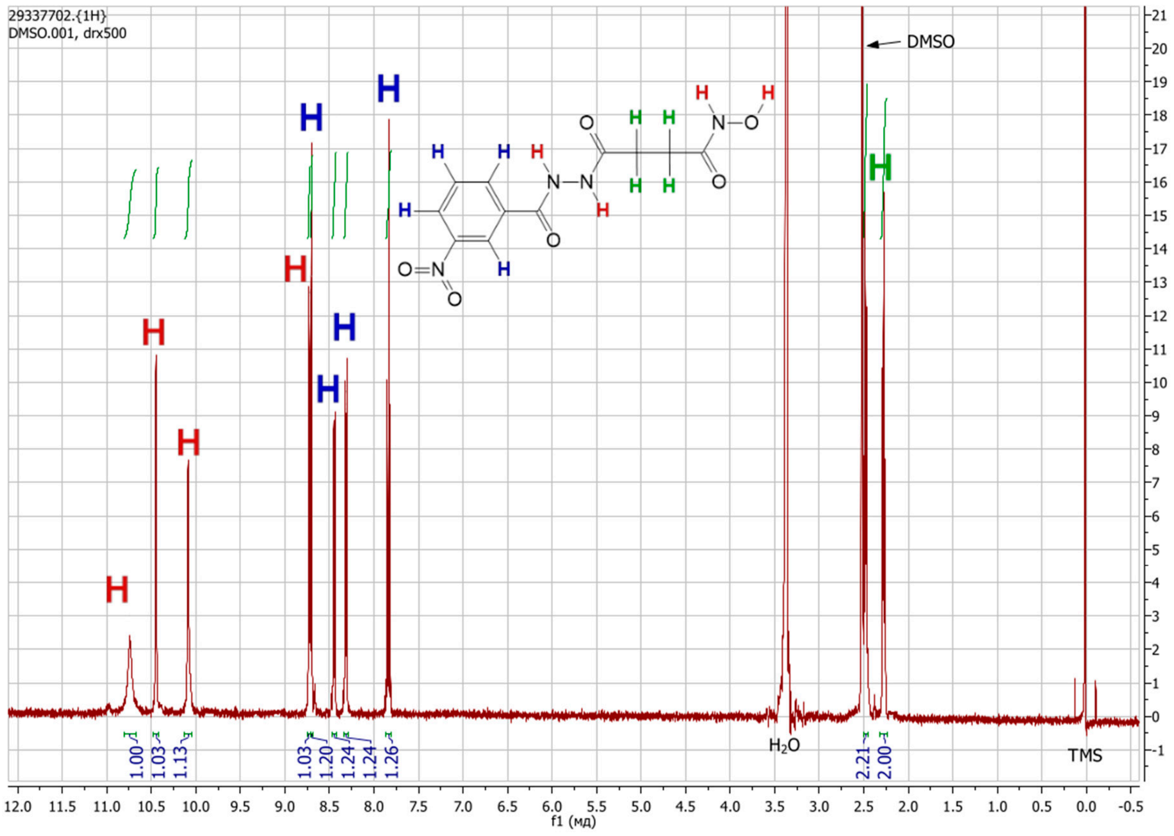

Figure S7 – ^{1}H NMR spectrum view of **2**

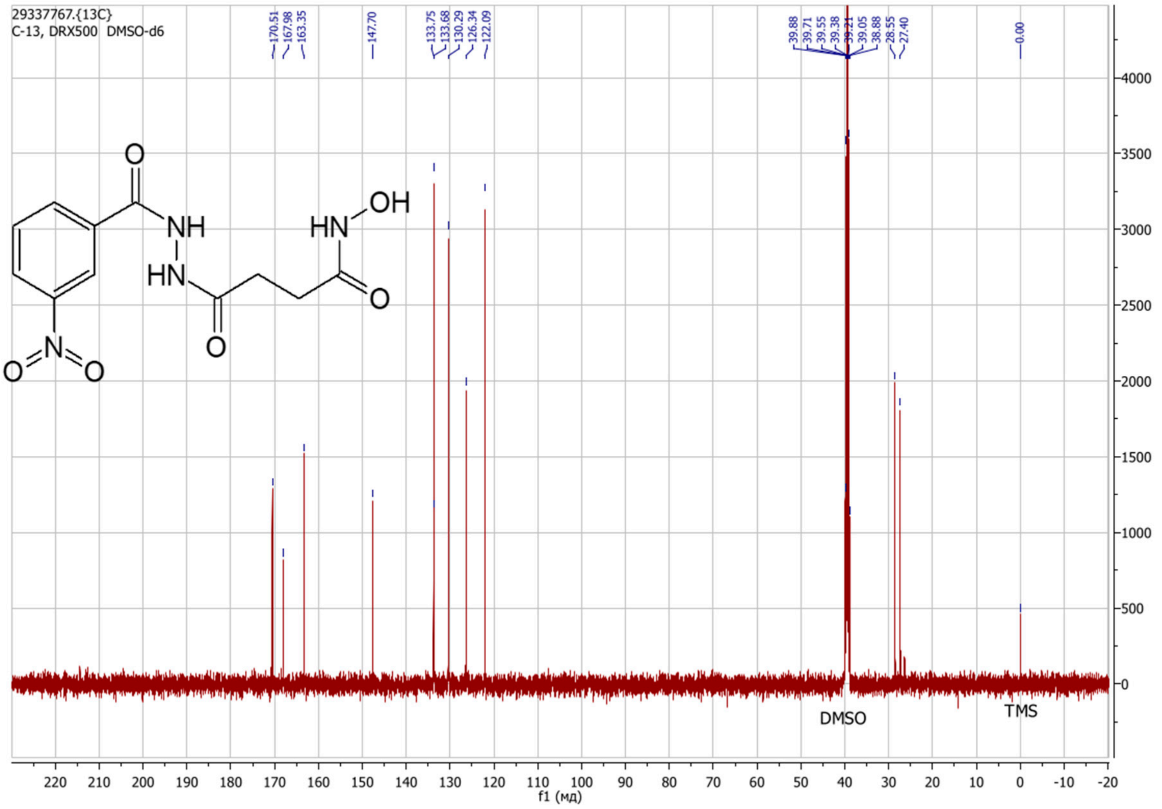

Figure S8 – ^{13}C NMR spectrum view of **2**

N-hydroxy-4-[2-(4-nitrobenzoyl)hydrazinyl]-4-oxobutanamide (3)

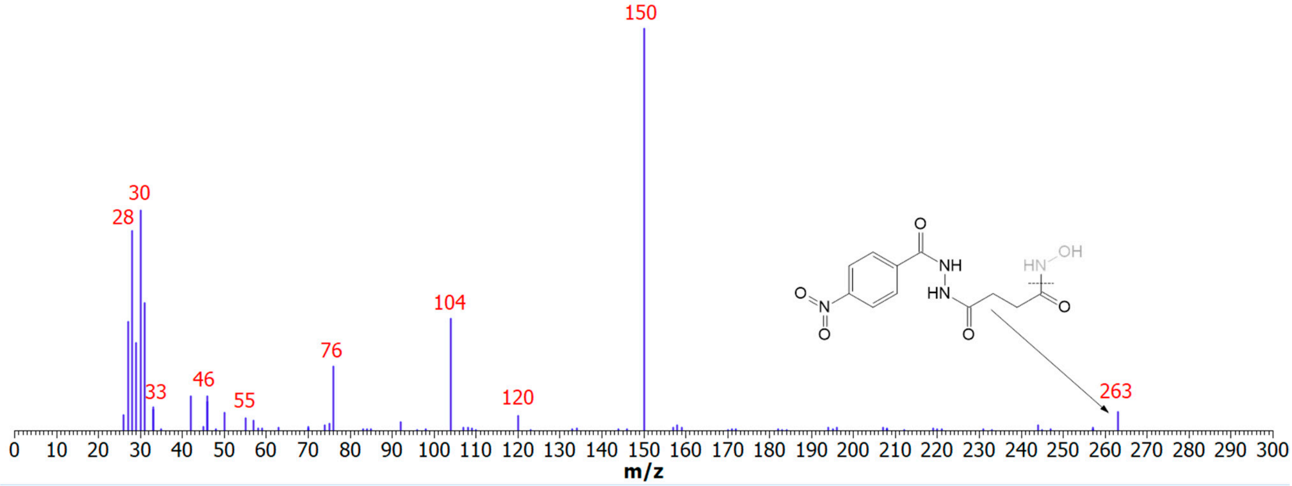

Figure S9 – Mass spectrum view of **3**

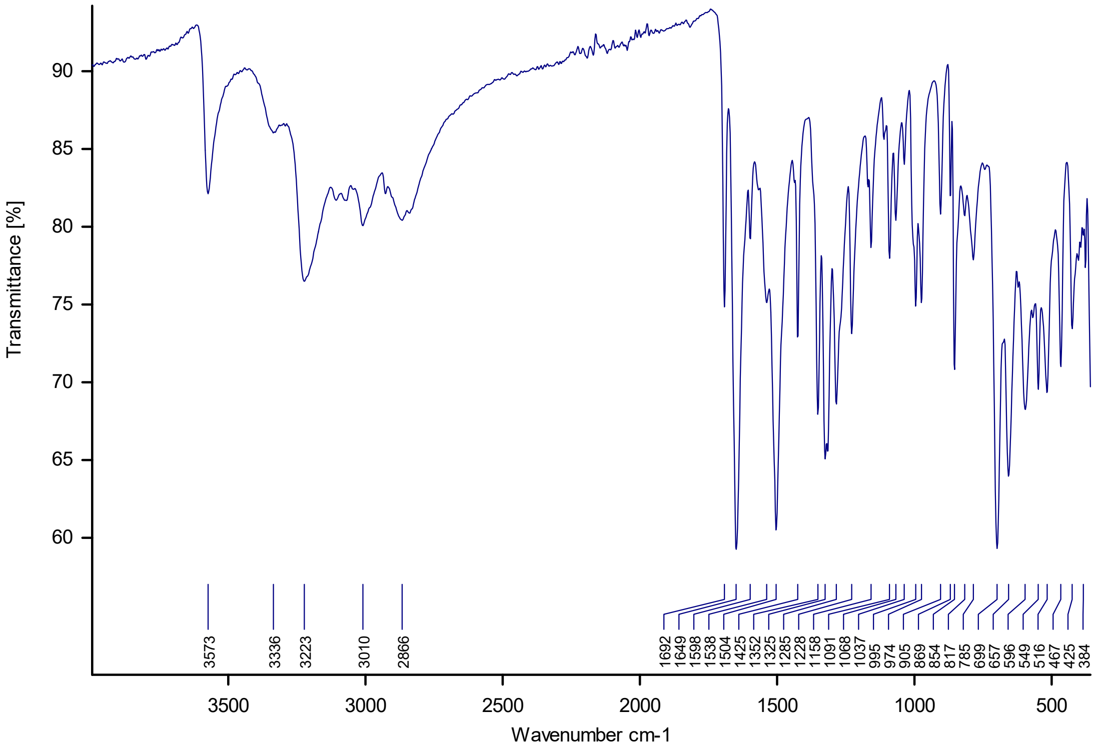

Figure S10 – IR spectrum view of **3**

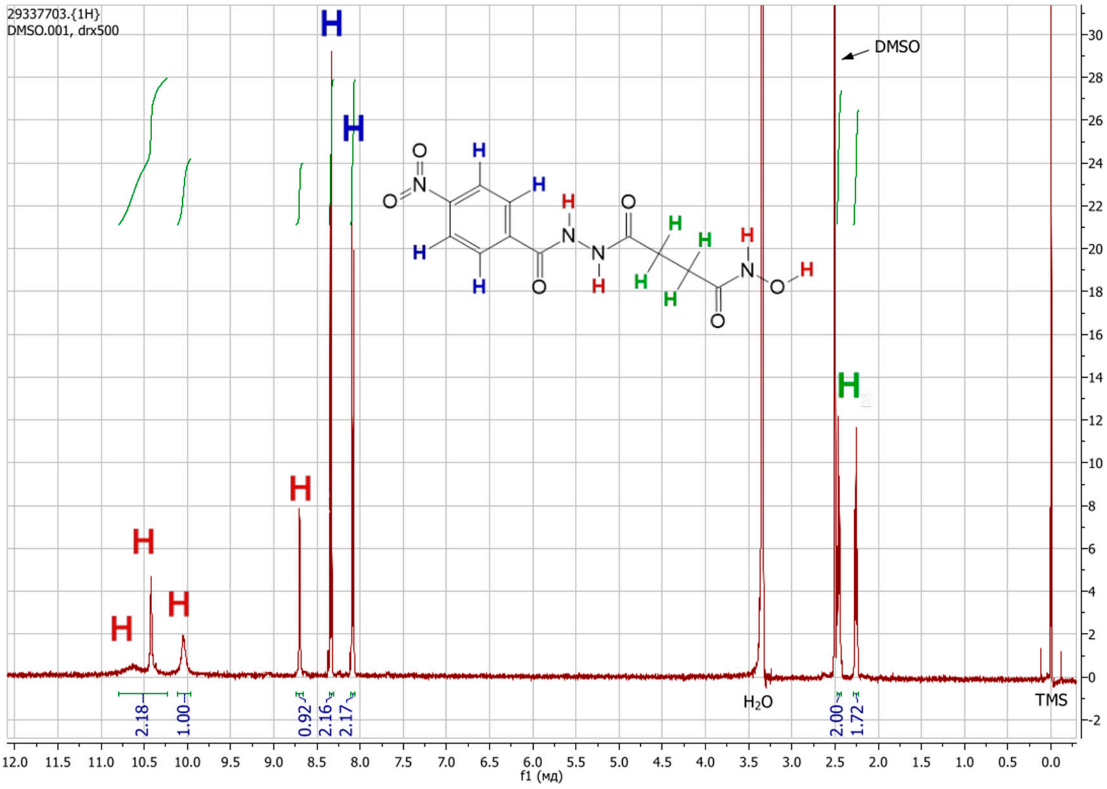

Figure S11 – ^{1}H NMR spectrum view of MMPI-3

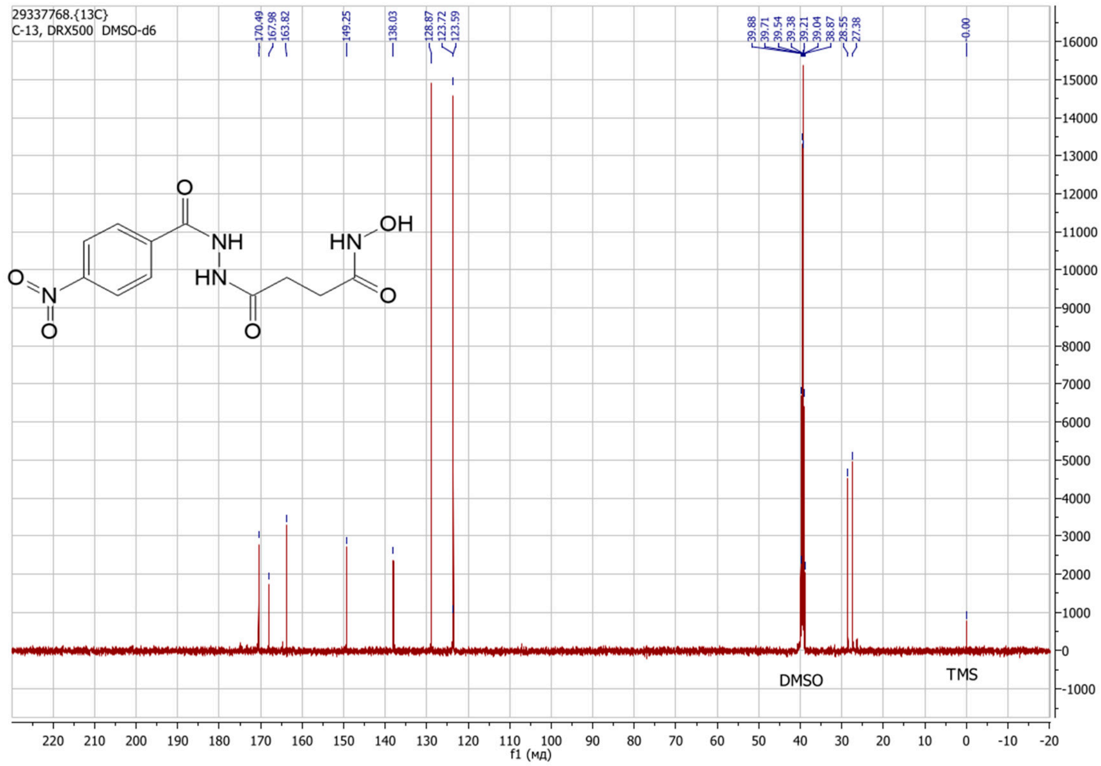

Figure S12 – ^{13}C NMR spectrum view of **3**

N^1-hydroxy-N^4-(4-iodophenyl)butanediamide (4)

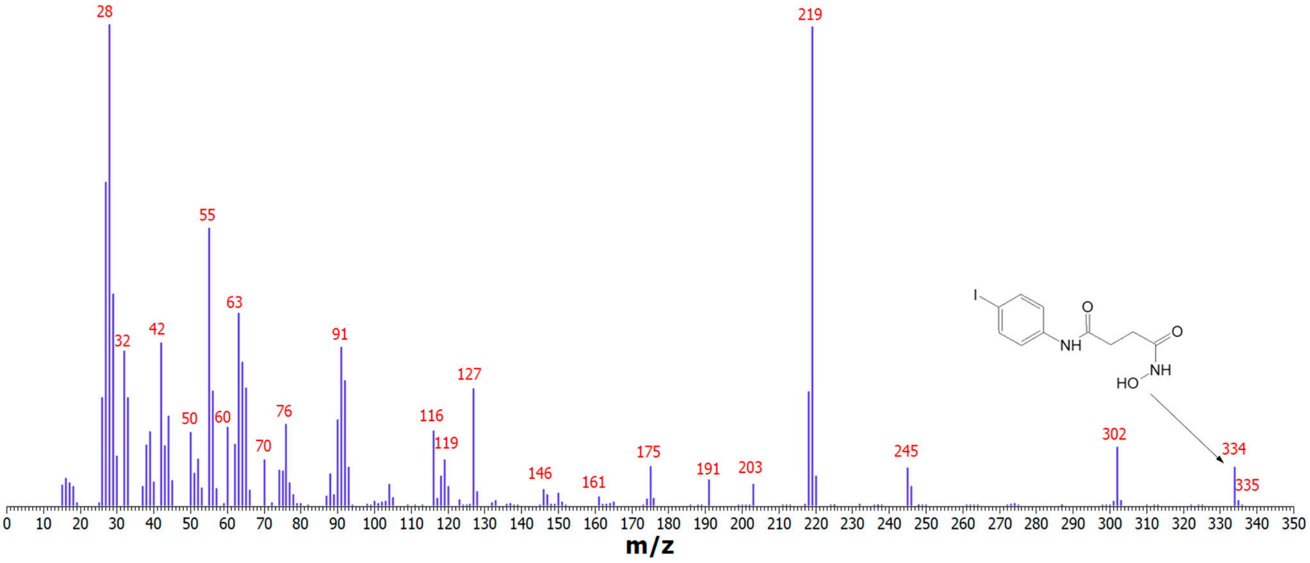

Figure S13 – Mass spectrum view of **4**

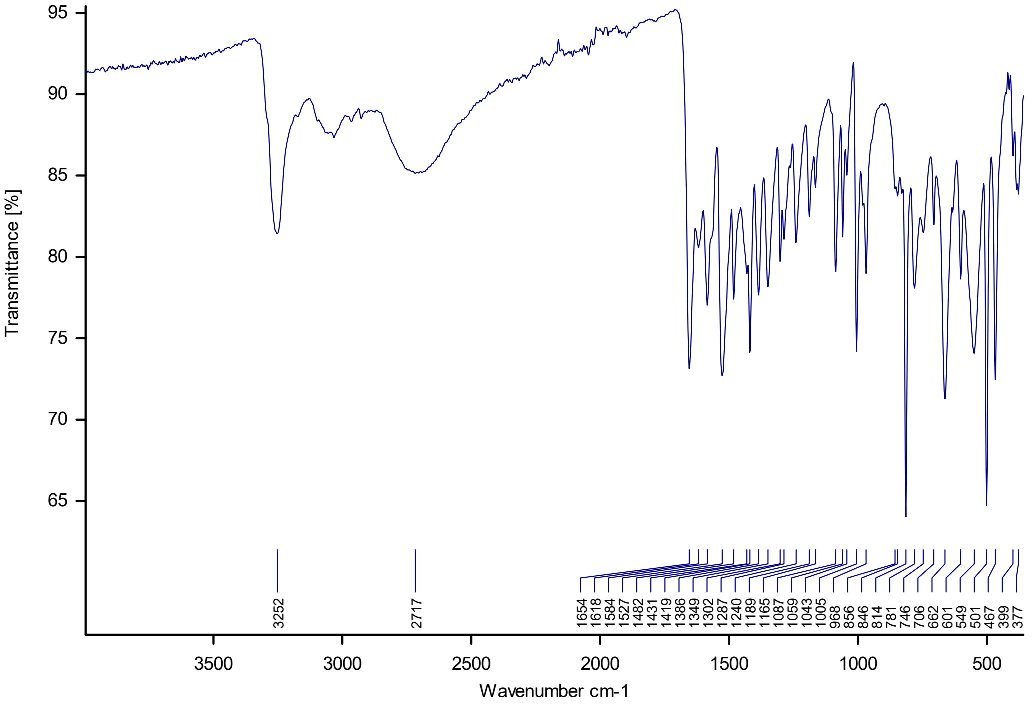

Figure S14 – IR spectrum view of **4**

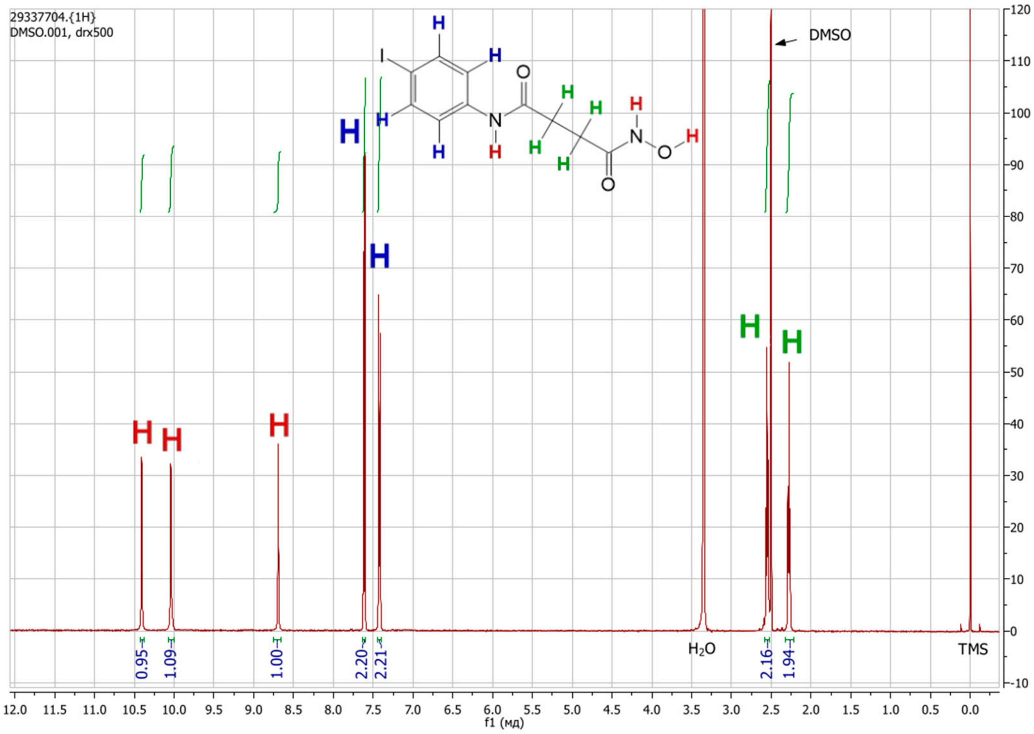

Figure S15 – ^{1}H NMR spectrum view of **4**

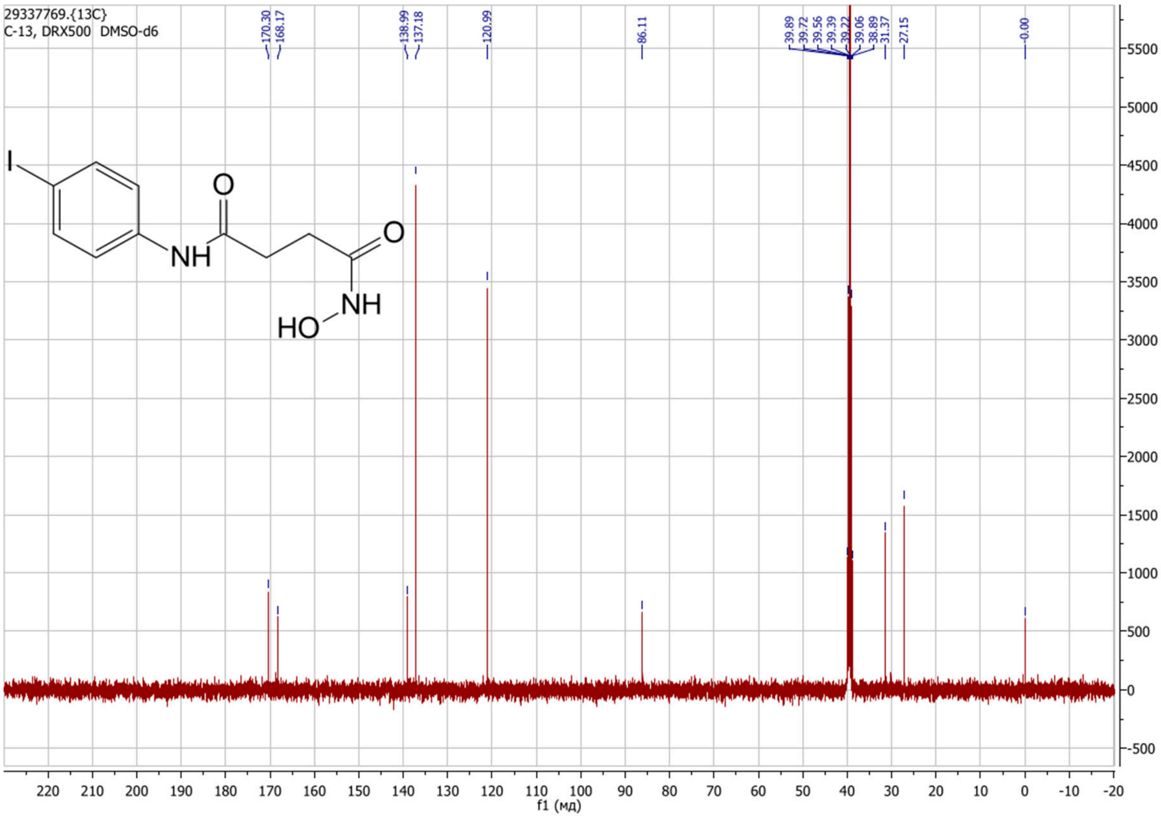

Figure S16 – ^{13}C NMR spectrum view of **4**

_N_-hydroxy-4-[2-(2-methoxybenzoyl)hydrazinyl]-4-oxobutanamide (5)

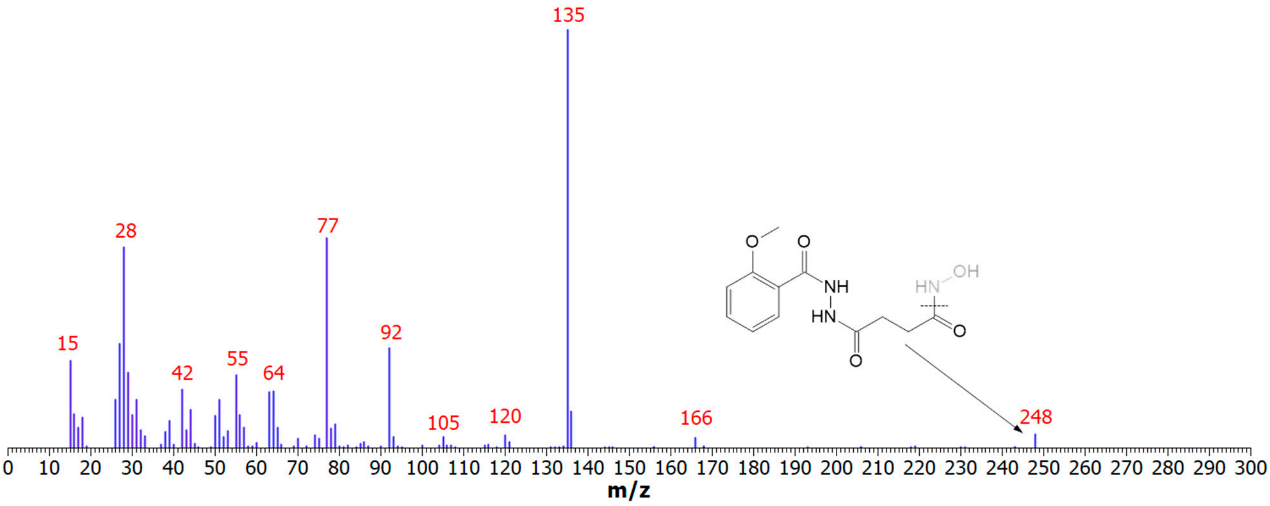

Figure S17 – Mass spectrum view of **5**

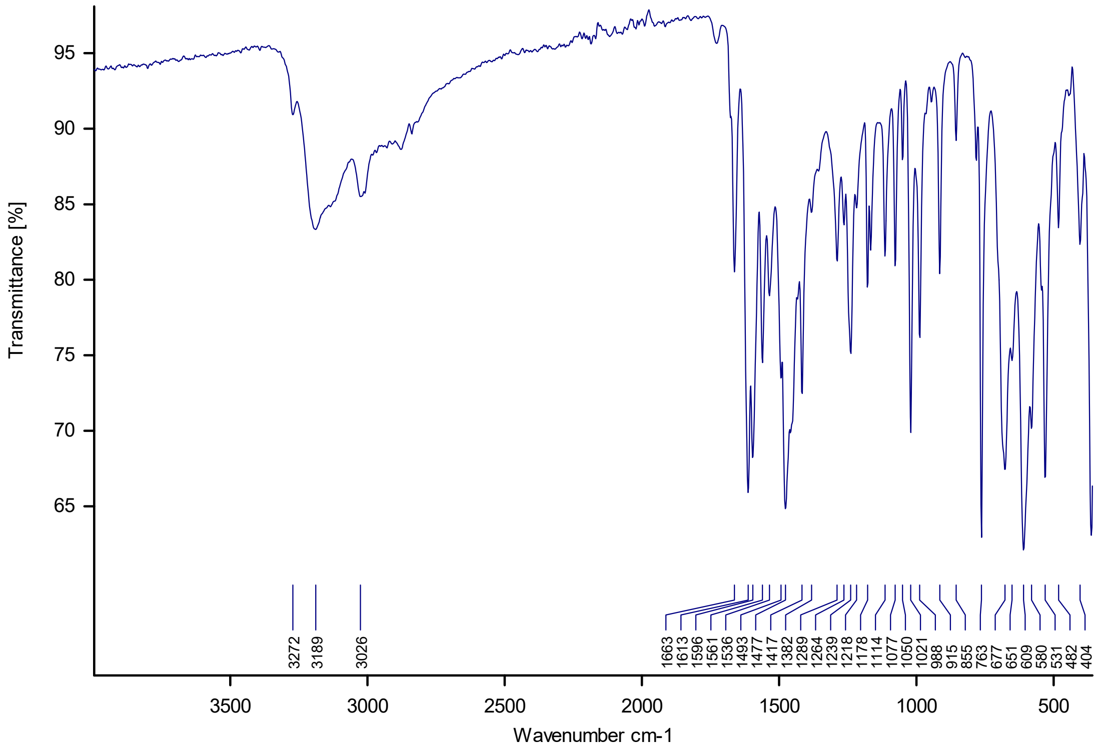

Figure S18 – Mass spectrum view of **5**

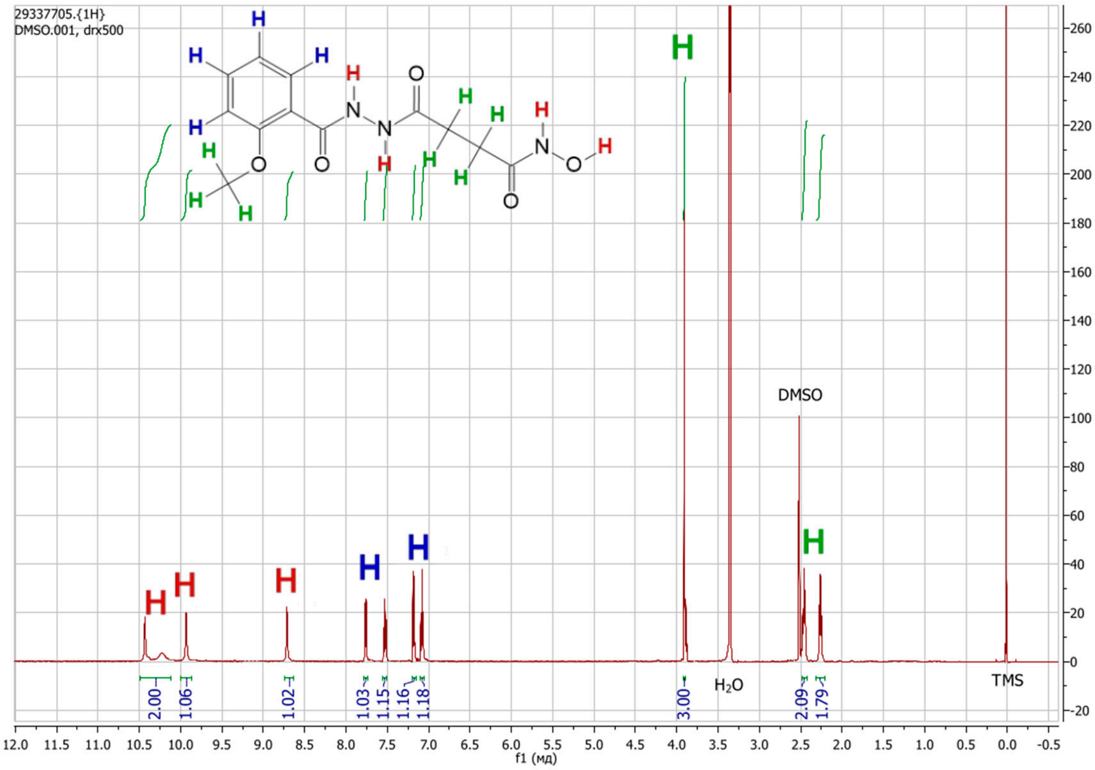

Figure S19 – ^{1}H NMR spectrum view of **5**

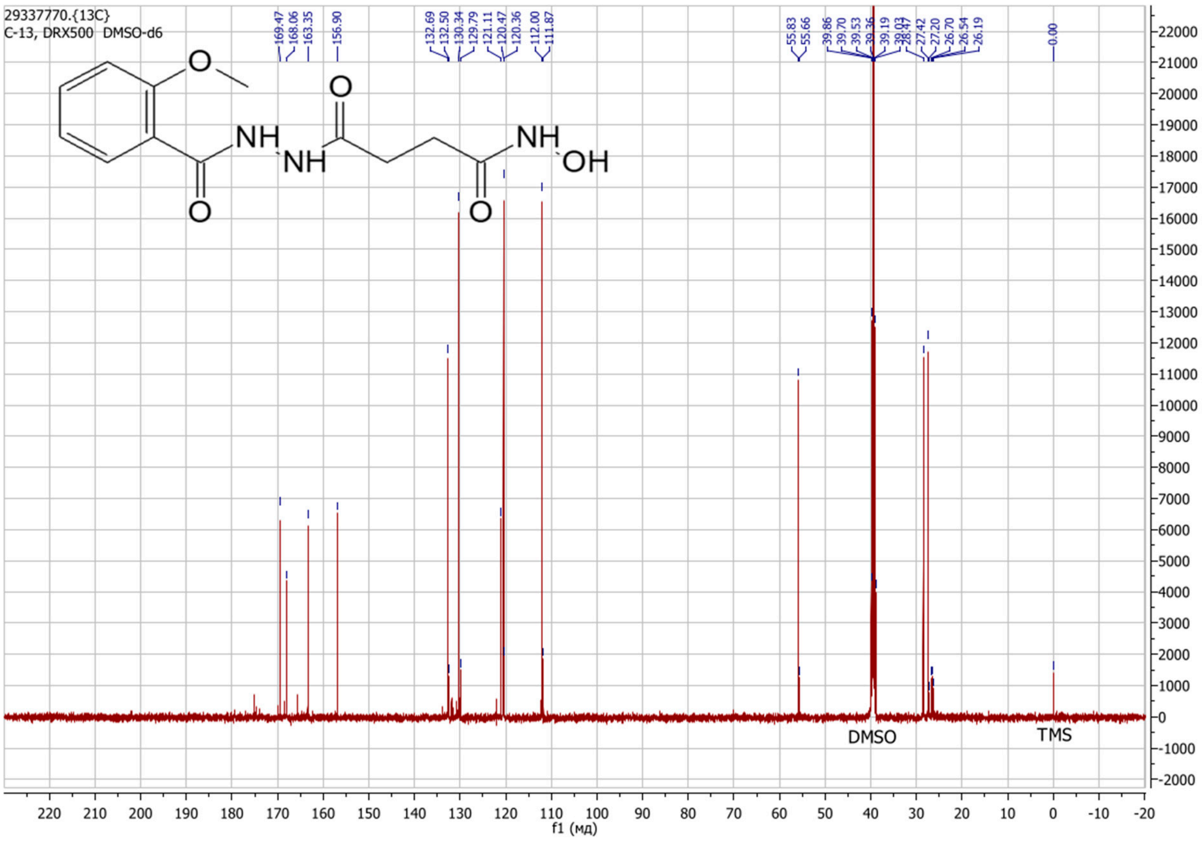

Figure S20 – ^{13}C NMR spectrum view of **5**
